# From Mechanochemically Driven Complexation and Multimodal Characterization to Stability and Toxicological Insight: A Study of Cinnarizine–β-Cyclodextrins Complexes

**DOI:** 10.3390/pharmaceutics17101338

**Published:** 2025-10-16

**Authors:** David Klarić, Lucija Kutleša, Mario Jug, Nives Galić

**Affiliations:** 1 Department of Chemistry, Faculty of Science, University of Zagreb, Horvatovac 102a, 10 000 Zagreb, Croatia; dklaric@chem.pmf.hr (D.K.); lkutlesa@chem.pmf.hr (L.K.); 2 Department of Pharmaceutical Technology, Faculty of Pharmacy and Biochemistry, University of Zagreb, A. Kovačića 1, 10 000 Zagreb, Croatia

**Keywords:** cinnarizine, cyclodextrins, mechanochemistry, solid-state characterization, *in vitro* permeability, *in vitro* dissolution, degradants, chemical stability, toxicity

## Abstract

**Background:** Cinnarizine (CIN) is a poorly soluble drug used in the treatment of vestibular disorders. Its solubility can be improved by complexation with cyclodextrins (CDs). This study focused on the preparation of 1:1 CIN/CD complexes with β-cyclodextrin (βCD) and its derivatives hydroxypropyl-β-cyclodextrin (HPβCD) and sulfobutylether-β-cyclodextrin (SBEβCD) by mechanical activation. **Methods:** Complexes were obtained under optimized grinding conditions using a high-energy vibrational mill with ZrO_2_ grinding media. Solid products were characterized by DSC, TGA, XRPD, and FTIR spectroscopy. Dissolution studies were performed in phosphate buffer (pH 4.5). The effect of βCD and HPβCD on CIN stability was assessed under hydrolytic (acidic, neutral, and basic) and oxidative conditions. A stability-indicating UHPLC-DAD-HRMS method was developed and validated, enabling CIN quantification in the presence of degradation products, whose structures were proposed based on HRMS/MS data. Potential toxicity, bioaccumulation, and mutagenicity of degradation products were predicted using QSAR modeling. Accelerated stability studies (40 °C, 75% RH) were conducted to evaluate long-term stability. **Results:** Solid-state analyses confirmed CIN/CD interactions in the ground products. The highest dissolution efficiency was observed for CIN/HPβCD complexes, while CD complexation did not alter CIN permeability in biomimetic membrane assays. CIN and its complexes demonstrated satisfactory chemical stability, with no degradation products detected under accelerated conditions. **Conclusions:** Solid-state complexes of CIN with CDs enhanced dissolution without compromising stability, supporting their potential as promising candidates for novel pharmaceutical formulations.

## 1. Introduction

**Figure 1:**
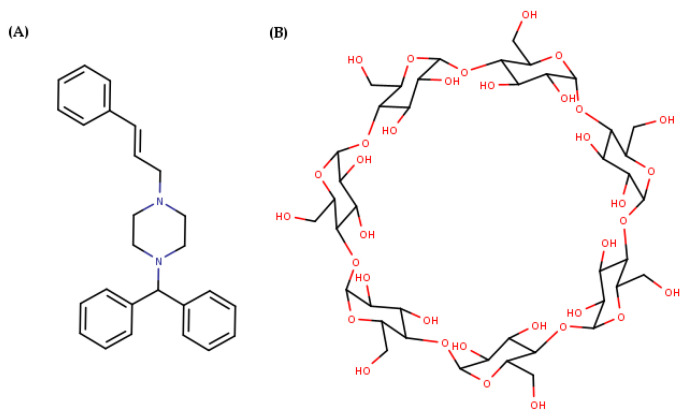
Structural formulas of (**A**) Cinnarizine and (**B**) β-cyclodextrin.

Cinnarizine (CIN) is a piperazine derivative (Figure 1) discovered by the Janssen Pharmaceutical Companies, exhibiting antihistaminic, antiserotonergic, antidopaminergic, and calcium channel-blocking properties. It is administered orally to treat vestibular disorders such as tinnitus, nausea, vomiting, and vertigo caused by Meniere’s disease and to prevent motion sickness. As CIN promotes cerebral blood flow, it may be beneficial in conditions such as cerebral thrombosis, cerebral embolism, cerebral arteriosclerosis, and diseases associated with poor peripheral circulation. CIN is also reported to be effective in treating some allergic diseases, such as chronic urticaria and senile skin pruritus [1,2]. Finally, recent studies highlighted CIN efficiency in migraine prophylaxis [3].

Chemically, CIN is a lipophilic weak base (log*P* = 5.6; p*K*a_1_ = 1.95 and p*K*a_2_ = 7.47), showing low aqueous solubility at pH values higher than 4.5. As a result, upon oral administration, CIN supersaturates and precipitates when it is transferred from a highly acidic environment in the stomach to the fasted small intestine, which limits its absorption in the gastrointestinal tract. The increased gastric acidity enhances the oral bioavailability of CIN in dogs and humans, and a positive food effect on CIN pharmacokinetics was also observed in healthy human volunteers when Arlevert^®^ (20 mg CIN tablets) were administered with a high-fat meal [4]. Because of that, different formulation approaches like polymeric dispersions [5], polymer-based crystallization inhibitors [6], cyclodextrin (CD) complexation [7,8,9], nanosponges [10] and lipid-based formulations [11,12,13] were considered to enhance the oral bioavailability of CIN. The use of CD-based technology appears to be particularly effective, providing a significant increase in CIN oral bioavailability [7,9,14]. CDs, as structurally related biocompatible cyclic oligosaccharides of a particular structure, can interact non-covalently with various sterically compatible drug molecules by supramolecular inclusion complex formation, thereby enhancing their solubility, stability, and bioavailability [15,16,17,18,19].

In previous studies, CD complexes of CIN were prepared using solution-based methods, such as co-precipitation, spray-drying [9], and freeze-drying [7]. However, such an approach requires the use of acidic solutions where CIN degradation might occur [20]. In other cases, organic solvents such as acetone and ethanol were added, which are challenging to remove completely and thus remain in the final product, impairing its biocompatibility and safety. In this sense, mechanochemical activation by grinding appears as a fast, highly efficient, convenient, versatile, sustainable, and eco-friendly method for enabling the preparation of CD complexes of CIN in the solid state without the use of any solvent [21]. In this instance, the primary aim of his study is to optimize the technological parameters of the grinding process in high-energy vibrational mills, considering the different grinding times and frequencies required to achieve successful CD complexation of CIN in the solid state. The complexes are prepared using parent β-cyclodextrin (βCD) and its chemically modified derivatives, hydroxypropyl-β-cyclodextrin (HPβCD) and sulfobutylether-β-cyclodextrin (SBEβCD). Changes in the residual drug crystallinity (RDC), monitored by differential scanning calorimetry (DSC) and X-ray powder diffraction (XRPD), along with Fourier-transform infrared (FTIR) spectroscopy, were used to track the solid-state interactions in the prepared products [22]. Furthermore, the functionality of the prepared complexes in providing the solubilization of CIN in the biorelevant media was assessed by *in vitro* dissolution studies to select the product most suitable for further formulation development. Finally, the effect of cyclodextrin complexation on CIN permeability was evaluated using a biomimetic membrane assay [23].

The chemical stability of drugs in pharmaceutical formulations is of great importance, as it enables the suitable shelf life of the dosage form and protects the drug from degradation in vivo upon administration. Earlier research suggests that the enhanced oral bioavailability of CIN upon coadministration with food may also be attributed to the enhanced chemical stability of the drug brought up by its interaction with the food components [4]. In this sense, the stabilizing effect of excipients used in formulation development is crucial. However, only a few papers deal with CIN stability under stress conditions based purely on decreasing CIN concentration, with no characterization of degradation products [22,23,24]. It was demonstrated that CIN remained stable under alkaline and thermal conditions but degraded under acidic and oxidative conditions. Very recently, liquid chromatography coupled to high-resolution mass spectrometry (LC-HRMS/MS) was used for the identification of two CIN degradation products formed under the stress conditions [25], one of them previously noticed during the accelerated and long-term storage conditions of the lipid-based CIN formulation [13]. The influence of β-CD and its derivatives on the CIN stability under stress conditions has not been systematically studied. To the best of our knowledge, only T. Tokumura et al. investigated the effect of β-CD on the degradation rate of CIN by UV/Vis spectroscopy [20].

Forced degradation experiments were conducted under oxidative, acidic, and basic conditions, and the resulting degradation products were characterized using ultra-high-performance liquid chromatography coupled with high-resolution mass spectrometry (UHPLC-HRMS), which enabled their structural elucidation. Finally, accelerated stability studies at 40 °C and 75% RH were performed to assess the stability of the co-ground products obtained to rationally select the most suitable CIN/CD complex for future formulation development.

## 2. Materials and Methods

Cinnarizine (CIN, 1-trans-cinnamyl-4-diphenylmethylpiperazine, CAS 298-57-7) was obtained from Biosynth s.r.o., Bratislava, Slovakia. β-cyclodextrin (βCD, CAS 7585-39-9), 2-hydroxypropyl-β-cyclodextrin (HPβCD, with an average degree of substitution, DS = 4.5, CAS 128446-35-5), and sulfobutylether sodium salt β-cyclodextrin (SBEβCD, DS = 6.5, CAS 182410-00-0) were obtained from CycloLab, Budapest, Hungary. A hydrochloric acid medium with a pH of 1.2 and the phosphate-buffer solution pH 4.5 were prepared according to the monograph 5.17.1. of the European Pharmacopoeia 11.0 [26]. Potassium bromide (KBr, CAS 7758-02-3) for spectroscopy (FTIR grade) was obtained from Acros Organics, Geel, Belgium. Methanol and formic acid, LC-MS grade, were purchased from Carlo Erba (Milano, Italy). Ultrapure water was obtained by Mili-Q Advantage A10 purification system (Merck, Darmstadt, Germany). All other chemicals were of p.a. grade and used as received.

### 2.1. Preparation and Characterization of Cyclodextrin Complexes in the Solid-State

Cyclodextrin complexes of CIN and the selected CDs were prepared at an equimolar drug-to-CD ratio using a high-energy vibrational mill (Mixer Mill MM 500 control, Retch GmbH, Haan, Germany) equipped with 50 mL grinding stations made of ZrO_2_. The batch size was 1 g, and the grinding was performed at 20 and 30 Hz for different periods using four ZrO_2_ grinding balls of 10 mm in diameter and four ZrO_2_ grinding balls of 5 mm in diameter. During the grinding, the system was thermostated at 10 °C. The samples prepared were collected in glass vials and stored at room temperature in a desiccator protected from the light until further analysis. The drug alone was subjected to the same treatment to assess the effect of grinding on the physicochemical properties of the drug.

Differential scanning analysis (DSC) was performed on a Discovery DSC 250 Differential Scanning Calorimeter (TA Instruments, New Castle, DE, USA). The samples were accurately weighed into aluminum pans (1–3 mg, Mettler M3 Microbalance, Gießen, Germany), sealed with pierced lids, and scanned under nitrogen purge of 50 mL/min over the temperature range of 25–200 °C employing a heating rate of 10 °C/min. The relative degree of drug crystallinity (*RDC*) in the analyzed samples was calculated according to Equation (1):

(1) $$RDC=\frac{{∆H}_{sample}}{{∆H}_{drug}}\times 100\%$$

where Δ*H*_sample_ and Δ*H*_drug_ are the fusion enthalpies of the CIN in the product (normalized to the drug content) and the pure drug, respectively. Measurements were carried out in triplicate, and the relative standard deviation of crystallinity data was <1.0%.

Thermogravimetric analysis (TGA) was performed on a Discovery TGA 550 thermogravimetric analyzer (TA Instruments, New Castle, DE, USA) employing a heating rate of 10 °C/min at a temperature range of 20–300 °C.

Powder X-ray diffraction (PXRD) patterns of the starting materials and co-ground products, placed on a silicon support, were recorded at room temperature using a Malvern Panalytical Aeris diffractometer of Bragg–Brentano geometry (Malvern Panalytical Ltd., Malvern, UK) equipped with a copper anode (CuKα; *λ* = 1.5406 Å), a Ni filter, and a PIXcel1D-Medipix3 detector. Data were collected in a continuous scan mode over the 2*θ* range of 5–50°, at a scanning speed of 0.022°/s, with a counting time of 18.87 s per step. The Kα_1_/Kα_2_ intensity ratio was 0.5, and the X-ray tube was operated at 40 kV and 15 mA.

For Fourier-transformed infrared spectroscopy (FTIR) analyses, samples and KBr powder were mixed at a 1:99 (*w*/*w*) ratio and compressed into a tablet. FTIR spectra were obtained using a Bruker Vector 22 spectrometer (Bruker Optics GmbH, Ettlingen, Germany) over the 4000–400 cm^−1^ range at 2 cm^−1^ resolution. Each spectrum represents the average of 32 scans recorded under ambient conditions and smoothed using the Savitzky–Golay algorithm (25-point window).

### 2.2. Stability of CIN During the Co-Grinding with CDs

To assess the stability of CIN during the co-grinding with CDs, 10 mg of the co-ground samples were weighed, transferred into 10 mL flasks, and dissolved in 1 mL of acetonitrile (ACN). After that, flasks were filled up to the mark with 0.1 M HCl, and the sample absorbance was measured at 253 nm employing a Carry 60 spectrophotometer (Agilent, Santa Clara, CA, USA) after suitable sample dilution with 10% ACN (*v*/*v*) in 0.1 M HCl. The validation parameters of the method are given in Table S1. The relative drug content for each sample was calculated as a ratio of the actual and theoretical drug content, expressed as a percentage.

### 2.3. In Vitro Dissolution Test

The mini paddle assembly of Agilent 708-DS Dissolution Apparatus (Agilent Technologies, Inc., Santa Clara, CA, USA), a scaled-down USP 2 apparatus, was used to mimic the fasted-state gastric dissolution conditions. A sample containing 37.5 mg of CIN was introduced into 125 mL of dissolution medium (hydrochloric acid, pH 1.2, or phosphate buffer, pH 4.5) maintained at 37 °C and stirred at 75 rpm. Aliquots of 5 mL were withdrawn at 5, 10, 15, 20, 30, 45, and 60 min and immediately replaced with an equal volume of fresh medium pre-thermostated at 37 °C to maintain a constant volume throughout the experiment. The collected samples were filtered through a Cromafil^®^ Xtra PES 20/25 membrane filter (Macherey-Nagel GmbH & Co. KG, Düren, Germany) and analyzed spectrophotometrically for drug content at 235 nm after appropriate dilution (Table S1). Filter compatibility was verified by a preliminary study. The test was performed in triplicate (CV < 5.0%) for each sample. A correction was applied for the cumulative dissolution caused by adding the fresh dissolution medium.

The dissolution efficiency (*DE*) parameter for the analyzed samples was calculated according to Equation (2):

(2) $${DE}_{60\;min}=\frac{\sum_{0}^{t}Qdt}{Q_{100\%}\times t}\times 100$$

where *Q* is the percentage of the dissolved drug and *t* is the examined dissolution time [27].

### 2.4. In Vitro Permeability Study

The *in vitro* permeability studies were performed employing the Phoenix dry heat vertical diffusion cells system (Teledine Labs, Chatsworth, CA, USA) with a nominal volume of the acceptor compartment of 15 mL and orifice diameter of 11.3 mm. The donor compartment was filled with 1.5 mL of 1.0 mM solution of the drug, free or as the selected co-ground complex with CD, in the hydrochloric acid medium, pH 1.2. Permeapad^TM^ biomimetic barriers (Innome Gmbh, Espelkamp, Germany) were placed between the donor and acceptor compartments. The acceptor compartment was filled with hydrochloric acid medium, pH 1.2, to circumvent the problems with the limited solubility of CIN at higher pH values and provide sink conditions. The system was thermostated at 37 °C. At preselected time points (i.e., 0.5, 1, 1.5, 2, 3, 4, 5 h) 400 μL were withdrawn from the acceptor compartment and immediately replaced with an equal volume of the same medium. Drug content in the collected samples was analyzed spectrophotometrically at 253 nm after suitable sample dilution. The permeability profiles were constructed by plotting the amount of the permeated drug over the surface area (*dQ*/*A*) as a function of time (*t*). The apparent permeability coefficient (*P_app_*) according to Equation (3):

(3) $$P_{app}=\frac{dQ}{dt}\times \frac{1}{A\times c_{0}}$$

where *c*_0_ is the initial concentration of the drug in the donor compartment. For each sample, the measurements were performed in triplicate.

### 2.5. Stability Testing of Prepared CIN/CD Complexes

#### 2.5.1. Forced Degradation Studies

A stock solution of CIN at 0.1 mg/mL, along with CIN/CD samples at 0.4 mg/mL, was prepared in 90% MeOH. Forced degradation studies were conducted under the following conditions: pure water, 1 M HCl, 1 M NaOH, and 0.3% H_2_O_2_, all at 80 °C for 8 h using a Lauda NB-D8/17 bath circulator (Lauda Scientific, Lauda-Königshofen, Germany).

#### 2.5.2. Accelerated Stability Studies

CIN samples (1 ± 0.25 mg) and co-ground CIN/CD samples (5 ± 0.50 mg) were weighed in triplicate, placed into Eppendorf tubes, and stored in a Memmert ICH100L eco stability chamber (Memmert, Schwabach, Germany) at 40 °C and 75% relative humidity. Samples were collected and analyzed at 0, 1, 2, and 3 months. Before analysis, each sample was dissolved in 0.75 mL of hydrochloric acid medium (pH 1.2), followed by the addition of 0.75 mL of methanol.

### 2.6. UPLC-DAD and UPLC-HRMS(/MS) Measurements

Stability-indicating UPLC-DAD (Agilent 1290 Infinity II) and UHPLC-HRMS (Agilent 6550 iFunnel Q-TOF) methods were developed and validated for the quantitative determination of CIN and for the structural characterization of its degradation products (Supplementary Materials, Tables S2 and S3). Analyses were performed on an Agilent ZORBAX RRHD Bonus-RP column (2.1 × 50 mm, 1.8 μm) under gradient elution with 0.1% formic acid in water (A) and methanol (B) at a flow rate of 0.20 mL/min. The injection volume was 1 μL, and detection of CIN was carried out at 252 nm. HRMS/MS analyses were conducted in positive ion mode, employing nitrogen as the collision gas. Prior to analysis, all samples were filtered through Chromafil^®^ PTFE-20/15 MS membrane filters (Macherey-Nagel GmbH & Co. KG, Düren, Germany).

### 2.7. Theoretical Toxicity Assessment

To predict *in vivo* acute toxicity (oral rat *LD*_50_, mg/kg), bioaccumulation factor (*BCF*), developmental toxicity (*DT*), and mutagenicity of CIN and its potential degradation products, the Toxicity Estimation Software Tool, T.E.S.T. (version 5.1.2.) from the U.S. Environmental Protection Agency (Washington, DC, USA) was used. T.E.S.T was operated in batch mode with toxicity values calculated by consensus method. Structures of CIN and its potential degradation products were imported in T.E.S.T. as files in MDL Molfile format. MarvinSketch version 25.3.0 was used for drawing, displaying and characterizing chemical structures, substructures and for generating SMILES strings (Chemaxon, Budapest, Hungary (https://www.chemaxon.com)).

### 2.8. Statistical Analysis

Data plotting and statistical analysis were performed using GraphPad Prism version 8.0.1. (GraphPad Software, Boston, MA, USA) and OriginPro 2016 (OriginLab Corporation, Northampton, MA, USA). Statistical comparisons between two groups were performed using Student’s *t*-test, while comparisons between multiple groups were performed using one-way ANOVA followed by Tukey’s multiple comparisons post hoc test. A *p*-value of ≤0.05 was considered to indicate statistical significance.

## 3. Results and Discussion

### 3.1. Preparation and Solid-State Characterization of Co-Ground CD Complexes

**Figure 2:**
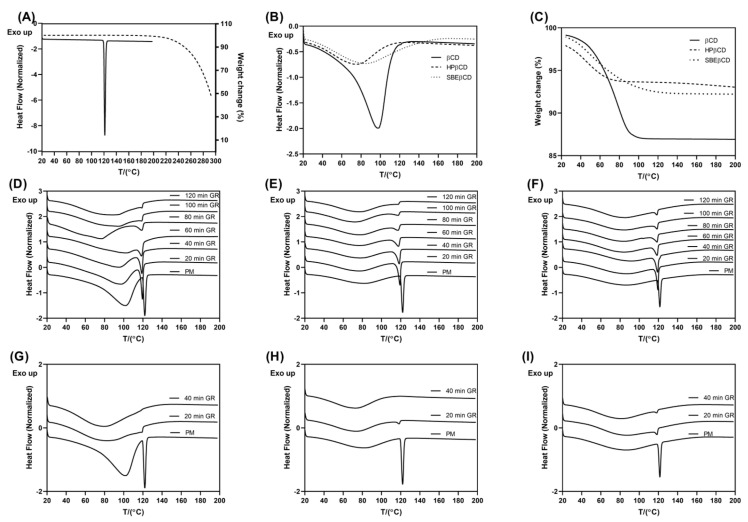
Thermograms of pure compounds and prepared co-ground samples: (**A**) DSC (solid line) and TGA (dotted line) curves of CIN; (**B**) DSC and (**C**) TGA curves of pure CDs; DSC curves of co-ground samples prepared by grinding at 20 Hz using (**D**) βCD, (**E**) HPβCD and (**F**) SBEβCD, respectively, processed for different time intervals up to 120 min; DSC thermograms of co-ground samples with (**G**) βCD, (**H**) HPβCD and (**I**) SBEβCD prepared by grinding at 30 Hz up to 40 min.

**Figure 3:**
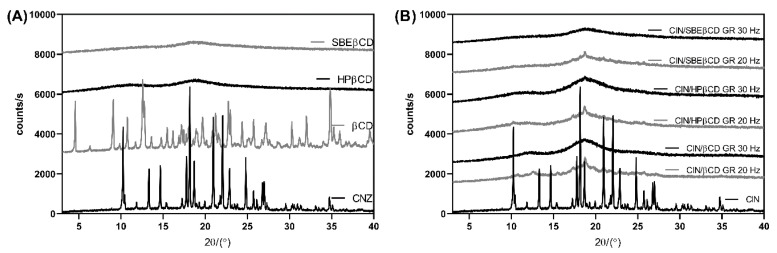
XRPD curves of (**A**) pure compounds and (**B**) CIN co-ground samples with CDs prepared at 20 and 30 Hz. The samples ground at 20 Hz were treated for 120 min, while the ones prepared at 30 Hz were ground for 40 min.

The technological parameters of the grinding procedure have a crucial impact on the solid-state interaction between the drug and selected CDs, thereby directing the performance of the co-ground products prepared. Grinding time and frequency are the most important variables, while their effect is closely related to the milling device employed and the drug/CD system treated [21,28,29]. In this study, grinding was performed at frequencies of 20 and 30 Hz for varying durations to determine the optimal conditions. The solid-state interactions in the prepared samples were monitored by DSC and XRPD, two complementary techniques routinely applied to characterize CD complexes, while TGA was employed to determine adequate experimental conditions for DSC analysis and to facilitate the interpretation of the DSC results [22]. The results obtained are presented in Figure 2 and Figure 3, respectively.

The DSC thermogram of CIN presented a sharp endothermic peak with an onset temperature of 119.87 °C, consistent with the melting point of the drug [1]. The TGA profile showed that the drug is thermally stable up to 180 °C, where its thermal decomposition began (Figure 2A). DSC thermograms of CDs presented broad dehydration peaks corresponding to the weight loss of 12.98, 4.19 and 6.57% for βCD, HPβCD and SBEβCD, respectively (Figure 2B,C). In the thermograms of CIN/CD co-ground mixtures, a decrease in the melting peak intensity and a slight shift to the lower temperatures can be observed as a function of the grinding time (Figure 2D–F, Table S4). When the grinding was performed at 20 HZ, the traces of the crystalline drug were still observed even after 120 min of grinding. The amount of the residual crystalline fraction was related to the type of CD used (Table S4). On the contrary, grinding at 30 Hz led to complete drug amorphization after 40 min treatment, except for the samples with SBEβCD, where the residual crystalline drug fraction was about 7.46% (Figure 2G–I). Prolonged CIN/SBEβCD sample grinding at 30 Hz leads to compaction, forming a solid mass that cannot be collected.

Further evidence on the solid-state interactions in the ground products was obtained by XRPD, a complementary technique routinely employed to verify the results obtained by thermal techniques. Unlike DSC, XRPD requires no sample pretreatment and may exclude thermally induced interaction between the drug and CD during the DSC scan [22]. XRPD diffractogram of CIN presented numerous sharp diffraction peaks at 10.52°, 13.43°, 14.91°, 18.41°, 18.89°, 21.16°, 22.29° and 27.03° consistent with the previous literature data, confirming the crystalline nature of the untreated drug [2,30]. βCD also presented a diffraction pattern typical of a crystalline compound, while HPβCD and SBEβCD showed a halo pattern typical of amorphous compounds (Figure 3). CIN/CD samples ground for 120 min at 20 Hz presented slight peaks typical of the drug, while those treated for 40 min at 30 Hz appeared completely amorphous, even in the case of CIN/SBEβCD. This may be attributed to the lower sensitivity of XRPD in detecting the crystalline phase in dominantly amorphous samples compared to DSC. Generally, conventional XRPD obtained using Bragg–Brentano optics cannot detect the crystalline drug phase when its content is below 5% [31].

Finally, to exclude the possibility of drug amorphization due to mechanical treatment, CIN was subjected to grinding at 30 Hz without adding CDs, and the samples obtained were analyzed by DSC. The results presented in Figure S1 and Table S5 clearly showed that the mechanical treatment alone led only to a slight reduction in the drug crystallinity, so the observed amorphization of the CIN in the co-ground samples with the CDs may be attributed to the successful drug/CD solid-state interactions obtained by grinding [21]. Such an amorphous solid phase allows greater molecular mobility and the establishment of inclusion and non-inclusion interactions between the CIN and CDs used [32].

**Figure 4:**
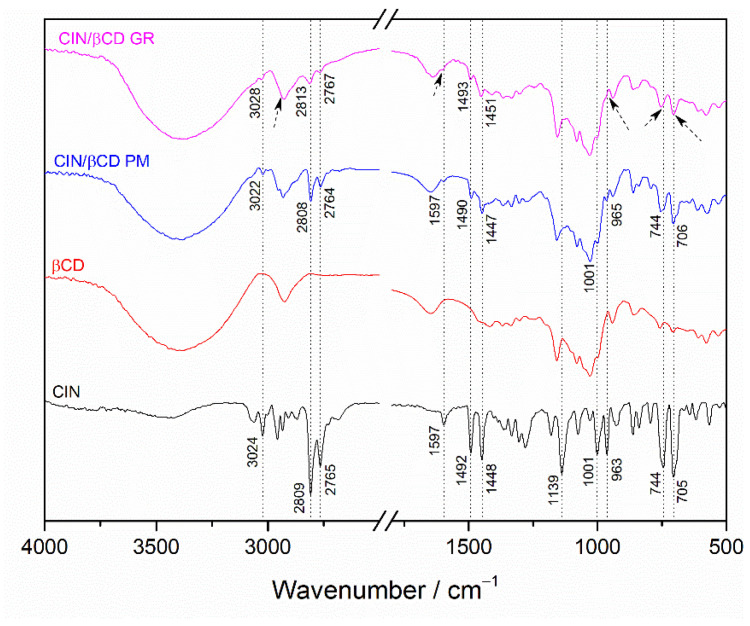
FTIR spectra of pure compounds CIN (–) and βCD (–), their physical mixture (–), and co-ground sample prepared at 30 Hz for 40 min (–).

The grinding process was further monitored using FTIR spectroscopy. Although FTIR analysis proved to be less informative than DSC and XRPD, several notable spectral changes provided evidence of solid-state interactions in the ground CIN/CD systems. The FTIR spectrum of the physical mixture closely resembled the superimposed spectra of the individual components (Figure 4). In contrast, the FTIR spectrum of the ground CIN/βCD sample exhibited a hypsochromic shift (indicated by the dotted line) in characteristic CIN bands [33,34,35,36] (Table S6), accompanied by a marked decrease in their intensities (indicated by arrows). Consequently, the FTIR spectrum of the ground CIN/βCD sample differed significantly from that of the corresponding physical mixture and more closely resembled the spectrum of pure βCD. Mechanochemical activation during grinding can promote amorphization and closer molecular contact between CIN and βCD, facilitating non-covalent interactions resulting in partial or complete inclusion of CIN molecules. These interactions may alter the local environment of the functional groups responsible for the characteristic CIN vibration, resulting in a decrease in band intensity. Additionally, the broad and intense nature of βCD bands, particularly in the O–H and C–H stretching regions, can easily obscure weaker CIN absorptions when the components are intimately mixed on a molecular level. According to Cannavà et al., such spectral changes are indicative of inclusion complex formation in the solid state [37]. Comparable spectral alterations were observed for the ground CIN/HPβCD and CIN/SBEβCD systems (Figures S2 and S3).

**Figure 5:**
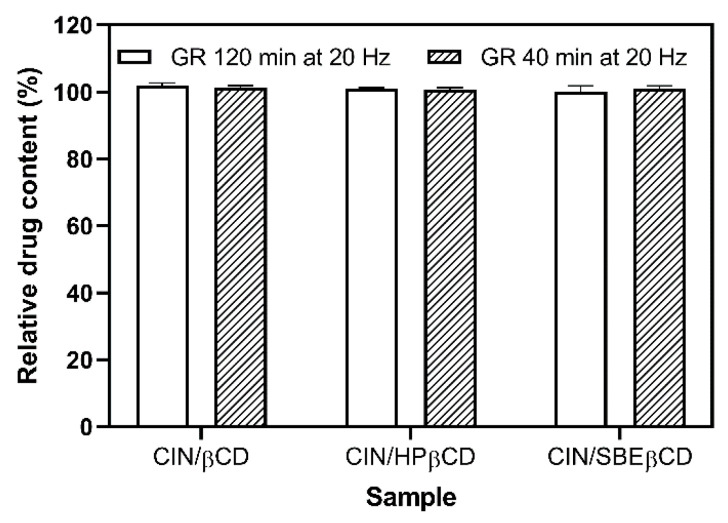
Relative drug content of the co-ground samples processed at 20 Hz for 120 min and 30 Hz for 40 min, respectively.

Prolonged grinding at elevated frequencies results in a high energy input to the treated system, potentially triggering the drug degradation or its unwanted interaction with the co-milling additive used, as demonstrated by Zanolla et al. in the case of praziquantel co-ground products with povidone and mesoporous silica [38,39,40]. Because of that, the chemical stability of the drug during the grinding procedure should be regularly monitored. In the case of CIN co-ground products with CDs tested, the drug content in each co-ground product prepared was expressed as a percentage of the theoretical value (i.e., relative drug content, Figure 5). The results showed no drug degradation, irrespective of the employed grinding parameters (i.e., grinding frequency and time) or the type of CD used, further emphasizing the suitability of grinding to prepare CD complexes of CIN.

### 3.2. In Vitro Dissolution and In Vitro Permeability Properties of the Co-Ground Complexes

**Figure 6:**
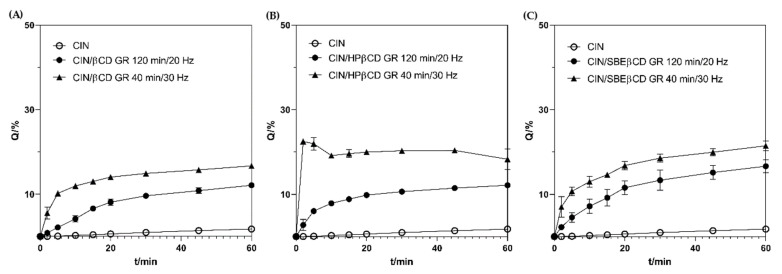
Comparison of *in vitro* dissolution profiles of CIN and (**A**) CIN/βCD, (**B**) CIN/HPβCD, and (**C**) CIN/SBEβCD co-ground samples in phosphate buffer pH 4.5 at 37 °C.

An *in vitro* dissolution test was performed to select the most suitable product for further drug formulation development. *In vitro* dissolution testing is widely used to mimic and predict in vivo performance of oral drug products in the gastrointestinal (GI) tract [41]. The FDA Dissolution Methods Database provides no recommendation on the dissolution methods for the oral products containing CIN [42]. When developing such a test, the dissolution medium composition and volume, apparatus type, and stirring rate should be carefully chosen to model the physiological environment within the GI tract [43]. The literature describes various methods for testing the dissolution/release of CIN from the formulations developed. They generally use paddle apparatus (USP 2) and hydrochloric acid medium pH 1.2 [2,5,44] or phosphate buffer solution with pH ranging from 4.5 to 6.8 [9,45,46,47]. In line with that, we have performed preliminary dissolution tests of the pure CIN employing both hydrochloric acid medium pH 1.2 and phosphate buffer medium pH 4.5. The test was performed using a mini paddle apparatus in conditions representing oral dose intake (i.e., 75 mg) with a glass of water (i.e., 250 mL). Although many standard dissolution tests employ a medium volume of 900 mL, this has limited in vivo relevance. In fact, the fasted human gastric volume after ingesting the dosage form with a glass of water is more likely to be 250 mL [43]. The results obtained (Figure S4) reveal fast CIN dissolution in the acidic medium, with the complete dose dissolved within 15 min, while the dissolution process at pH 4.5 was slow and incomplete, limited by the low drug solubility in those conditions. Therefore, we opted to perform *in vitro* dissolution testing at pH 4.5 to critically assess the functionality of the complexes prepared. The *in vitro* profiles of CIN and their co-ground complexes with CDs are presented in Figure 6.

To facilitate the comparison of the dissolution enhancement achieved by each of the co-ground complexes, the *in vitro* dissolution efficiency parameter was calculated [27], and then the dissolution enhancement factor was obtained as the ratio between the dissolution efficiency of the complex and that of the pure drug, expressed as a percentage (Table 1). The co-grinding with CDs significantly enhanced the *in vitro* dissolution properties of CIN at pH 4.5 (*p* < 0.0001), regardless of the applied grinding frequency. When comparing the performance of the products ground at 20 Hz, CD type did not statistically affect the dissolution properties of the product (*p* > 0.05). As demonstrated by DSC analysis, samples prepared by grinding at 20 Hz contained notable amounts of residual drug fraction (Table S4), indicating only partial drug/CD interaction. Because of that, the effect of CD on CIN dissolution was hampered by incomplete drug complexation. On the contrary, products prepared by grinding at 30 Hz always performed better than those prepared at 20 Hz, using the same CD type (*p* < 0.001). This may be attributed to a higher degree of drug amorphization and consequent more intense drug/CD solid state interactions, as discussed previously. Among products obtained at 30 Hz, the enhancement in the dissolution followed the line βCD < SBEβCD < HPβCD (Table 1). Parent βCD often appears as the least potent dissolution enhancer, while the highest dissolution efficiency was observed for the complex with HPβCD. This contrasts with the previous results reported by Järvinen et al. [7], which showed a higher affinity of CIN for SBEβCD than HPβCD, and consequent better solubilization of CIN with SBEβCD. The observed discrepancy may be attributed to the different substitution degrees of SBEβCD used in those studies, as well as the inability of grinding to provide complete amorphization of CIN under the applied conditions.

However, the supersaturation phenomenon might be observed for CIN/HPβCD complex ground at 30 Hz (Figure 6). Tanaka et al. [48] in an *in vivo* animal study showed that CIN precipitates in the duodenum after gastric emptying and thereafter the precipitate is rapidly re-dissolved and absorbed in the upper and middle intestine. The rapid re-dissolution was attributed to a small particle size of the precipitate and the presence of the bile salts. Therefore, it might be presumed that the case of CIN/HPβCD GR 30 Hz product, the presence of HPβCD would further enhance this process, thus contributing to the fast and complete drug absorption in vivo, as demonstrated previously for CIN/CD physical mixture [14] or lyophilized complex [7].

Further evidence in this regard was obtained by the *in vitro* permeability studies employing biomimetic membranes constructed as a sandwich of two cellulose-hydrate membranes enclosing a layer of dry phospholipids between them. Such a structure provides good storage stability and high robustness against extreme pH values and solvents like dimethyl sulfoxide and ethanol, providing a suitable model for permeability testing in early formulation development [49]. Under these conditions, the apparent permeability coefficients (*P_app_*) for CIN and CIN/HPβCD were determined to be (1.32 ± 0.13) × 10^−5^ and (1.32 ± 0.07) × 10^−5^, respectively, showing that the CD complexation had no significant effect on the CIN permeability (*p* > 0.05).

### 3.3. Effect of Cyclodextrins on CIN Stability

The stability of active pharmaceutical ingredients and their formulations is a key factor in maintaining their safety and therapeutic effectiveness. Studies have demonstrated that β-cyclodextrins can affect a drug’s chemical stability, either enhancing it or, in some cases, reducing it [16,50,51]. Accordingly, as a continuation of our study and based on the solid-state characterization and *in vitro* dissolution results, we investigated the stability of pure CIN, as well as its complexes with βCD and HPβCD.

A stability-indicating UHPLC-DAD-HRMS method was developed and validated for the quantitative analysis of CIN in the presence of its degradation products, as well as for the structural characterization of those degradation products.

#### 3.3.1. Forced Degradation Studies

Forced degradation studies are a primary approach for anticipating stability issues, developing stability-indication methods, and identifying degradation products and their formation pathways. Samples of pure CIN and its complexes with βCD and HPβCD were subjected to hydrolytic (acid, neutral, and basic) and oxidative degradation at 80 °C.

**Figure 7:**
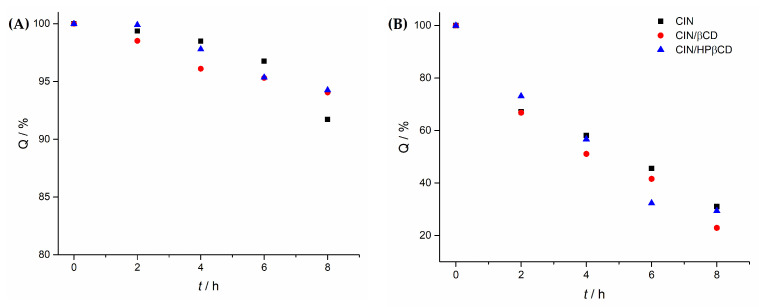
Forced degradation studies on pure CIN and its complexes with βCD and HPβCD in (**A**) 1 M HCl, and (**B**) 0.3% H_2_O_2_ at 80 °C.

Cinnarizine, whether in its pure form or complexed with βCD and HPβCD, has been found to be susceptible to degradation under acidic conditions, and particularly under oxidative stress (Figure 7).

#### 3.3.2. Identification of Cinnarizine Degradation Products

Today, LC-MS is regarded as an essential technique for structural characterization of minor components, such as impurities and degradation products, in drug substances and formulations [52,53]. Time-of-flight mass spectrometry (TOFMS) platforms equipped with electrospray ionization (ESI) sources represent the state-of-the-art in LC-HRMS analyses for impurities and degradation products. Although ESI is classified as a “soft” ionization technique, it is susceptible to in-source fragmentation (ISF). This phenomenon arises from the significant pressure differential that ions encounter as they transition from the atmospheric pressure region into the low-pressure environment of the mass analyzer [54]. Nonselective ISF can complicate mass spectral interpretation by generating additional fragment ions, which may obscure the identification of key peaks. In severe cases, the molecular ion signal of the analyte may not be detectable at all. To address this issue, researchers have focused on optimizing in-source parameters in LC-MS systems to minimize the impact of ISF and improve spectral clarity [54]. However, ISF phenomenon has demonstrated significant utility for detecting and identifying unknown compounds in targeted [55], non-targeted metabolomics [56,57,58] and environmental sample analysis [59].

**Figure 8:**
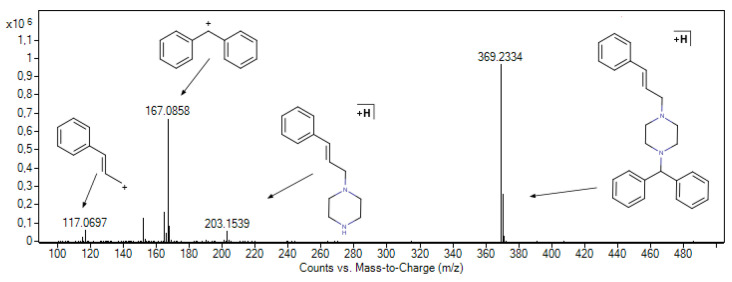
HRMS spectrum of CIN showing its characteristic ISF signals.

As a piperazine derivative, CIN contains two labile N–C bonds, which lead to multiple ISF signals in its MS^1^ spectrum (Figure 8). The monoprotonated molecular ion [M+H]^+^ was detected at *m*/*z* 369.2334 (exact mass: 369.2325; Δ 2.37 ppm). Characteristic ISF signals were observed at *m*/*z* 203.1539 (exact mass: 203.1543), 167.0858 (exact mass: 167.0855), and 117.0697 (exact mass: 117.0699), corresponding to the [C_13_H_19_N_2_]^+^, [C_13_H_11_]^+^, and [C_9_H_9_]^+^ fragment ions, respectively. Fragment ions generated through ISF are often similar, though not necessarily identical, to those produced by conventional MS/MS fragmentation via collision-induced dissociation (CID). MS/MS spectra obtained from CID experiments on above mentioned ions can be found in Supplementary Materials (Figures S5–S8, Table S7).

Observed ISF signals served as markers to identify the retention times of unknown CIN degradation products (DPs) by extracting their ion chromatograms (EICs) from the total ion chromatogram (TIC) of the sample exposed to forced degradation. This approach was based on the strategy introduced by Liu et al. [59], who demonstrated that characteristic ISF signals can be effectively used to flag and track related degradation products within complex LC-MS datasets.

**Figure 9:**
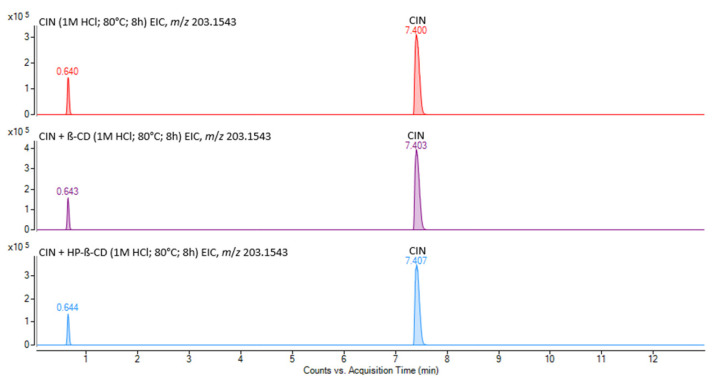
EICs of *m*/*z* 203.1543 marker ion for the CIN (–), CIN/βCD (–), and CIN/HPβCD (–) samples exposed to acidic forced degradation.

Extraction of ion chromatograms for the *m*/*z* 167.0855 marker in samples subjected to acidic forced degradation showed no additional DP peaks, apart from the CIN peak observed at a retention time (rt) of 7.4 min. In contrast, the EICs for the *m*/*z* 117.0697 and 203.1543 markers (Figure 9) revealed the presence of degradation product DP1 in all three samples, appearing at rt of 0.6 min. Interestingly, in the mass spectra at the given rt (Figure S9) ion at *m*/*z* 203 was assigned to the monoprotonated molecular ion of the DP1 after performing MS/MS experiments (Figures S10 and S11, Table S8). This DP, cinnamyl piperazine, was already described by Hassan et al. [60].

**Figure 10:**
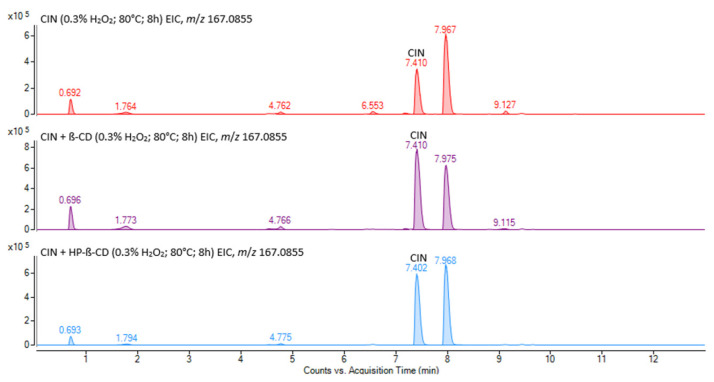
EICs of *m*/*z* 167.0855 marker ion for the CIN (–), CIN/βCD (–), and CIN/HPβCD (–) samples exposed to oxidative forced degradation.

Extraction of ion chromatograms for the *m*/*z* 203.1543 marker in samples subjected to oxidative stress revealed no additional degradation product peaks, aside from the CIN peak at the retention time of 7.4 min. In contrast, the EICs for the *m*/*z* 117.0697 marker (Figure S12), and especially for *m*/*z* 167.0855 (Figure 10), indicated the presence of several degradation products.

Both *m*/*z* 117.0697 and 167.0855 markers were detected at a retention time of 0.7 min, suggesting that the corresponding degradation product (DP2) retained both of these structural fragments from the cinnarizine molecule. However, when compared to the spectra of the blank samples, only the ion at *m*/*z* 233.1308 was newly observed (Figure S13). CID experiments on this ion (Figures S14 and S15, Table S9) showed fragmentation exclusively to *m*/*z* 117.0697, while the *m*/*z* 167.0855 fragment was absent. This indicates that the *m*/*z* 167.0855 marker likely originates from a co-eluting degradation product whose molecular ion was not detected in this study. A similar case was observed for degradation products eluting at 1.8 and 9.1 min, where only the *m*/*z* 167.0855 signal was present in the corresponding mass spectrum. At a retention time of 0.8 min, the presence of both *m*/*z* 117.0697 and a signal at *m*/*z* 233.1303 suggests the formation of a structural isomer of DP2, referred to here as DP3 (Figure S16). CID analysis revealed similar fragmentation patterns for both DP2 and DP3 (Figure S17), and both degradation products appear to contain two hydroxyl groups on the piperazine moiety. The marker ion *m*/*z* 167.0855 was detected at a retention time of 4.7 min, along with the ion at *m*/*z* 253.1708, which was assigned to the monoprotonated molecular ion [M+H]^+^ of degradation product DP4 (Figure S18). CID analysis (Figures S19 and S20, Table S10) identified this compound as 1-benzhydrylpiperazine, a well-known cinnarizine impurity and metabolite [60,61,62,63]. The *m*/*z* 167.0855 marker was also observed at retention times of 6.6, 7.2, and 8.0 min, while the *m*/*z* 117.0697 marker was detected only at 6.6 and 8.0 min. These fragments, together with the ion at *m*/*z* 385.2316 (Figure S21), correspond to the most abundant degradation product, DP5, eluting at 8.0 min. Its structure was previously proposed by Jain and Khan as *N*-oxide based on MS as well as ^1^H and ^13^C NMR data [25]. MS/MS-based structural characterization (Figures S22 and S23, Table S11) suggests that these degradation products contain a single hydroxyl group on the piperazine moiety, although its exact position cannot be determined solely from MS data. At 8.1 min, DP5 appears to co-elute with a minor degradation product, DP6, which is also associated with both *m*/*z* 117.0697 and 167.0855 markers. The ion at *m*/*z* 401.2250 (Figure S24) was assigned to the [M+H]^+^ molecular ion of DP6, suggesting a doubly hydroxylated structure. The same ion was also observed at retention times of 6.6 and 7.2 min (Figures S25 and S26), indicating the presence of structural isomers of DP6. MS/MS analysis of the ion at *m*/*z* 401 at 6.6 min (DP7), 7.2 min (DP8), and 8.1 min (DP6) helped clarify the absence of the *m*/*z* 117.0697 marker at 7.2 min. While DP6 and DP7 showed similar fragmentation patterns (Figures S27–S29, Tables S12 and S13), fragmentation of the molecular ion corresponding to DP8 produced a signal at *m*/*z* 295 but lacked the *m*/*z* 117 fragment entirely (Figure S30, Table S14). This suggests that degradation of the cinnamyl moiety occurred in DP8, likely due to epoxidation (Figure S31). The presence of βCD or HPβCD did not lead to the formation of any additional degradation products of CIN in any of the forced degradation studies. However, among the tested systems, the formation of above-mentioned degradation products was least pronounced in the forced degradation study of the CIN/HPβCD complex, compared to both pure CIN and the CIN/βCD system.

Among the detected degradation products, only DP5 can be characterized as a major CIN degradation product. The relative amount of DP5 accounted for approximately 69% of total degradation products in pure CIN sample, 75% in the CIN/βCD system, and 66% in the CIN/HPβCD system. All other detected degradation products were present in minor amounts, each representing less than 1% of the total degradation profile. The amount of the degradation product was estimated semi-quantitatively from UPLC peak areas. The concentration of the degradation product was estimated by assuming equal detector response at the wavelength of 252 nm for the degradation product and the parent drug. The values are therefore approximate and reported as estimated percentages.

#### 3.3.3. *In Silico* Toxicological Profiling of Cinnarizine Degradation Products

To assess the potential toxicity of CIN degradation products, acute oral toxicity (rat *LD*_50_), bioaccumulation factor, developmental toxicity, and mutagenicity were predicted using quantitative structure–activity relationship (QSAR) modeling via the T.E.S.T. software. For degradation products formed under oxidative forced degradation, where the exact site of hydroxylation could not be definitively established based on MS data, all plausible structural isomers were included in the toxicity evaluation (Table S15).

The evaluation of the toxicity of CIN and its potential degradation products formed during the forced degradation studies reveals a notable variation in toxicological profiles. Cinnarizine itself exhibits a moderate acute toxicity with an oral *LD*_50_ value of 433.47 mg/kg in rats, while its degradation product, formed under acidic stress, cinnamyl piperazine (DP1), shows increased toxicity (*LD*_50_ = 329.11 mg/kg), suggesting greater acute effects. Similar values are calculated for 1-benzhydrylpiperazine (DP4), which also shows increased toxicity (*LD*_50_ = 347.30 mg/kg). Most other degradation products generally demonstrate significantly higher *LD*_50_ values (e.g., DP2/DP3 = 984.17 − 1706.30 mg/kg), indicating lower acute toxicity. However, it is noteworthy that several possible structures of DP5 and DP8 exhibit higher predicted toxicity levels compared to CIN itself. These structures correspond to *N*-oxide derivatives, which is particularly interesting in the case of DP5, as its *N*-oxide structure was previously proposed by Jain and Khan, as previously mentioned [25]. Additionally, all of the DPs exhibit reduced bioaccumulation potential (lower *BCF* values) compared to the parent compound. With respect to developmental toxicity (DevTox), the majority of CIN DPs exhibit significantly lower predicted values compared to the parent compound. Notably, most of the proposed structures of DP6 and DP7 show elevated developmental toxicity (DevTox > 0.8), exceeding that of CIN, which may warrant further investigation. Interestingly, although the *N*-oxide derivatives of DP5 and DP8 display lower *LD*_50_ values, indicating higher acute toxicity, their developmental toxicity levels are actually lower than cinnarizine’s. In contrast, other possible isomeric forms of these degradation products demonstrate higher developmental toxicity. Mutagenicity predictions are generally low across all structures, with most DPs scoring below 0.6, suggesting limited genotoxic risk. Overall, while the majority of CIN DPs are less acutely toxic and less bioaccumulative, isolated cases of elevated developmental toxicity highlight the importance of comprehensive toxicological screening in stability and safety assessments.

#### 3.3.4. Accelerated Stability Studies

**Figure 11:**
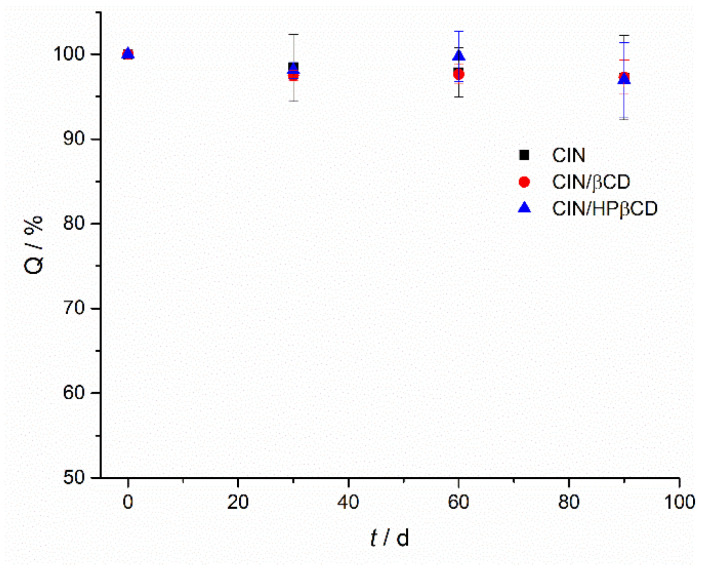
CIN content in samples of pure CIN, CIN/βCD and CIN/HPβCD samples prepared by grinding during the accelerated stability studies for 3 months.

Pure CIN was not prone to degradation under accelerated stability conditions. Both co-ground systems CIN/βCD and CIN/HPβCD showed satisfying chemical stability as well. The recovered CIN content at the end of the 3-month testing was 92–102% of the initial content in all cases (Figure 11).

No DPs were detected in any of the samples analyzed by UHPLC-HRMS after three months of storage in stability chambers. The monitoring was performed using the same strategy applied in the forced degradation studies, based on ISF markers and the [M+H]^+^ ion signals of previously characterized DPs. None of these markers or signals were observed in the stability samples.

## 4. Conclusions

Grinding Cinnarizine with CDs produced amorphous powdered products, with the extent of CIN amorphization depending on both the CD derivative used and the grinding conditions. All co-ground CIN/CD complexes demonstrated markedly improved *in vitro* dissolution in phosphate buffer at pH 4.5, while CIN permeability *in vitro* remained unaffected. Forced degradation studies of CIN and its co-ground CD complexes revealed several drug-related degradants, with some showing slightly higher predicted toxicity values than the parent compound. However, the CIN complexes with βCD and HPβCD exhibited satisfactory stability under accelerated conditions, without formation of previously characterized degradants. These findings highlight βCD and HPβCD as promising carriers for the further pharmaceutical development of CIN formulations.

## Figures and Tables

**Table 1:** Dissolution efficiency parameter and the dissolution enhancement factor for CIN and its co-ground complexes in phosphate buffer pH 4.5 thermostated at 37 °C.

Sample	Dissolution Efficiency/(%)	Enhancement Factor
CIN	0.89 ± 0.04	-
CIN/βCD GR 20 Hz	8.32 ± 0.50	9.33 ± 0.56
CIN/βCD GR 30 Hz	13.88 ± 0.14	15.56 ± 0.16
CIN/HPβCD GR 20 Hz	9.73 ± 0.25	10.91 ± 0.29
CIN/HPβCD GR 30 Hz	19.74 ± 0.44	22.13 ± 0.50
CIN/SBEβCD GR 20 Hz	11.85 ± 0.70	13.28 ± 1.90
CIN/SBEβCD GR 30 Hz	16.95 ± 0,96	19.00 ± 1.07

## Data Availability

The original contributions presented in the study are included in the article/Supplementary Materials; further inquiries can be directed to the corresponding author.

## Supplementary Materials

The following supporting information can be downloaded at: https://www.mdpi.com/article/10.3390/pharmaceutics17101338/s1, Table S1: The validation parameters of the UV/VIS method employed for the quantification of CIN in the obtained samples; Table S2: Chromatographic parameters for stability-indicating UPLC-DAD and UPLC-HRMS methods; Table S3: Instrument parameters for acquiring total ion chromatograms; Table S4: Melting temperature, fusion enthalpy and relative drug crystallinity of the CIN samples co-ground with CDs at 20 and 30 Hz for different time intervals; Figure S1: DSC thermograms of CIN ground at 30 Hz up to 60 min; Table S5: Melting temperature, fusion enthalpy and relative drug crystallinity of the CIN sample ground at 30 Hz for different time intervals; Table S6: Assignation of FTIR spectra of CIN, CIN/βCD physical mixture, and ground sample prepared at 30 Hz for 40 min; Figure S2: FTIR spectra of pure compounds CIN and HPβCD, their physical mixture, and co-ground sample prepared at 30 Hz for 40 min; Figure S3: FTIR spectra of pure compounds CIN and SBEβCD, their physical mixture, and co-ground sample prepared at 30 Hz for 40 min; Figure S4: *In vitro* dissolution profiles of CIN in hydrochloric acid medium, pH 1.2, and phosphate-buffer solution, pH 4.5, at 37 °C; Figures S5–S8, Table S7: HRMS characterization of CIN; Figures S9–S31, Tables S8–S14: Forced degradation studies; structural characterization of CIN degradation products by HRMS; Table S15: Proposed structures and toxicity data for CIN degradation products.
