# Supplementary material for: From Mechanochemically Driven Complexation and Multimodal Characterization to Stability and Toxicological Insight: A Study of Cinnarizine–β-Cyclodextrins Complexes
Source: Pharmaceutics. 2025 Oct 16;17(10):1338. doi: 10.3390/pharmaceutics17101338 (PMC12567510; doi:10.3390/pharmaceutics17101338)
Supplement: Supplementary file 1 [file pharmaceutics-17-01338-s001.zip › pharmaceutics-3912435-supplementary.pdf]

pharmaceutics

Article

From Mechanochemically Driven Complexation and Multimodal Characterization to Stability and Toxicological Insight: A Study of Cinnarizine–β-Cyclodextrins Complexes

David Klarić [1], Lucija Kutleša [1], Mario Jug [2,*], and Nives Galić [1,*]

[1] Department of Chemistry, Faculty of Science, University of Zagreb, Horvatovac 102a, Zagreb, Croatia; dklaric@chem.pmf.hr (D.K.); lkutlesa@chem.pmf.hr (L.K.)
[2] Department of Pharmaceutical Technology, Faculty of Pharmacy and Biochemistry, University of Zagreb, A. Kovačića 1, Zagreb, Croatia;
* Correspondence: mjug@pharma.hr (M.J.); ngalic@chem.pmf.hr (N.G.)

Supporting information datasheet

Contents

Supporting information datasheet 1
1. Materials and methods 1
2. Characterization of CIN complexes in solid state 2
2.1. DSC analyses 2
2.2. FTIR spectroscopy analyses 3
2.3. *In vitro* dissolution assessment 5
3. Effect of β-cyclodextrins on CIN chemical stability 5
3.1. Hydrolytic acidic degradation 8
3.2. Oxidative degradation 10
4. *In silico* toxicological profiling of CIN degradation products 0

 https://doi.org/10.3390/pharmaceutics17101338

pharmaceutics

1. Materials and methods

Table S1. The validation parameters of the UV/VIS method employed for the quantification of CIN in the obtained samples.

Solvent system	λ_{max} / (nm)	a	b	r^2	LOD / (µg mL^{-1})	LOQ / (µg mL^{-1})	working range (µg mL^{-1})
10% ACN in 0.1 M HCl	253	0.0503	0.0025	1.0000	0.03	0.11	0.20 – 22.00
10% ACN in phosphate buffer pH 4.5	253	0.0535	0.0066	0.9999	0.06	0.19	0.20 – 22.00

LOD – limit of detection. LOQ – limit of quantification.

Table S2. Chromatographic parameters for stability-indicating UPLC-DAD and UPLC-HRMS methods.

Parameter	Developed LC method		
Column	Agilent ZORBAX RRHD Bonus-RP		
Mobile phase A (MF_A)	0.1% (*v*/*v*) formic acid in water		
Mobile phase B (MF_B)	0.1% (*v*/*v*) formic acid in methanol		
	Time / min	MF_A / %	MF_B / %
Gradient elution profile	0	95	5
	10 – 13	5	95
Injection volume (UHPLC-HRMS)	1 – 5 µL		
Injection volume (UHPLC-DAD)	2 µL		
Mobile phase flow	0.20 mL/min		
Column temperature	40 °C		
Detection (DAD)	210, 252, 265, and 275nm		
Detection (HRMS, ESI+)	ESI+ (100 – 1300 *m*/*z*)		

Table S3. Instrument parameters for acquiring total ion chromatograms, TICs.

Parameter	Value
Sheath gas temperature	250 °C
Sheath gas flow	11 L/min
Nebulizer pressure	25 psi
Capillary voltage	2500 V
Nozzle voltage	1000 V
Drying gas temperature	200 °C
Drying gas flow	17 L/min
Fragmentor voltage	100 V

2. Characterization of CIN complexes in solid state

2.1. DSC analyses

Table S4. Melting temperature (T_{onset}), fusion enthalpy (ΔH_f) and relative drug crystallinity (*RDC*) of the CIN samples co-ground with CDs at 20 and 30 Hz for different time intervals.

Sample	Grinding frequency / (Hz)	Grinding time / (min)	T_{onset}/°C	ΔH_f/(Jg^{-1})	*RDC*/(%)
CIN/βCD	20	0	119.87	25.17	93.48
		20	116.88	18.22	67.25
		40	115.44	13.84	51.03
		60	114.1	10.30	38.03
		80	112.37	8.11	29.95
		100	109.89	5.64	20.82
		120	116.23	1.99	7.34
CIN/HPβCD	20	0	119.78	21.07	91.80
		20	116.11	15.14	65.59
		40	114.3	10.20	44.20
		60	111.83	8.69	37.68
		80	111.17	5.31	23.08
		100	110.55	3.27	14.17
		120	109.61	2.13	9.21
CIN/SBEβCD	20	0	119.53	15.99	99.40
		20	116.87	13.01	80.85
		40	115.49	13.01	80.87
		60	113.81	8.98	55.80
		80	114.76	5.31	32.98
		100	115.6	2.45	15.23
		120	115.7	2.23	13.85
CIN/βCD	30	0	119.87	25.17	93.48
		20	112.55	2.7696	10.22
		40	-	0	0.00
CIN/HPβCD	30	0	119.78	21.07	91.80
		20	115.22	1.51	6.53
		40	-	0	0
CIN/SBEβCD	30	0	119.53	15.99	99.40
		20	115.17	2.88	17.86
		40	115.24	0.59	3.72

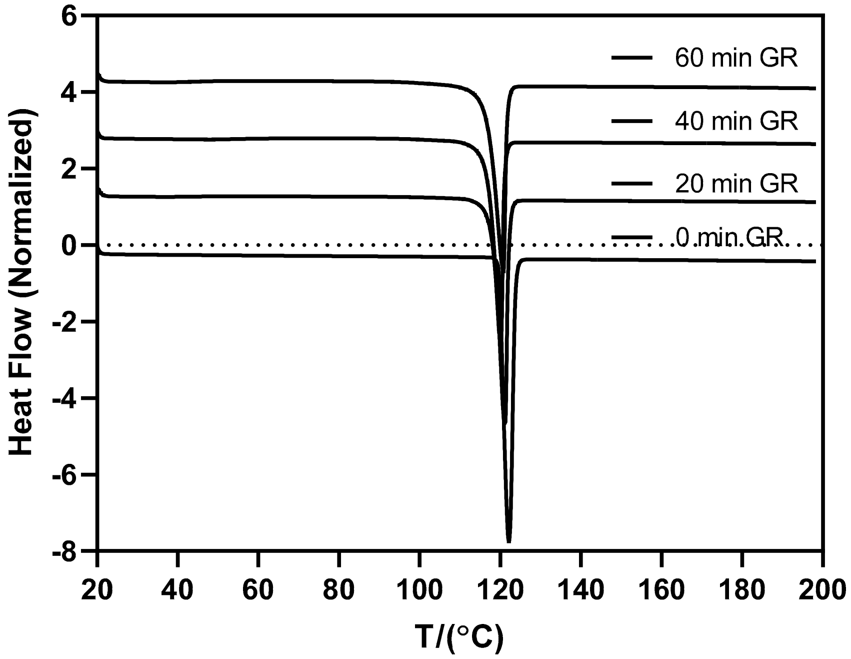

Figure S1. DSC thermograms of CIN ground at 30 Hz up to 60 minutes.

Table S5. Melting temperature (T_{onset}), fusion enthalpy (ΔH_f) and relative drug crystallinity (*RDC*) of the CIN sample ground at 30 Hz for different time intervals.

Sample	Grinding frequency / (Hz)	Grinding time / (min)	T_{onset}/°C	ΔH_f/(Jg^{-1})	*RDC*/(%)
CIN	30	0	119.69	110.52	100.00
		20	117.86	97.99	88.67
		40	117.06	97.26	88.00
		60	116.35	96.94	87.71

2.2. FTIR spectroscopy analyses

Table S6. Assignation of FTIR spectra of CIN, CIN/βCD physical mixture (PM), and ground sample (GR) prepared at 30 Hz for 40 min.

Wavenumber / cm^{-1}			Assignation
CIN	CIN/βCD PM	CIN/βCD GR	
3024	3022	3028	ν(C–H)$_{arom.}$
2809	2808	2813	ν(–N–(CH_3)$_2$)$_{aliph.}$
2765	2764	2767	ν(–N–(CH_3)$_2$)$_{aliph.}$
1597	1597	-	ν(C=C)$_{arom.}$
1492	1490	1493	ν(C=C)$_{arom.}$+δ(C–H)$_{arom.}$
1448	1447	1451	
1139	Masked by βCD	-	ν(C–N)
1001	1001	-	δ(=C–H)
963	965	-	δ(=C–H)$_{arom.}$
744	744	-	
705	706	-	

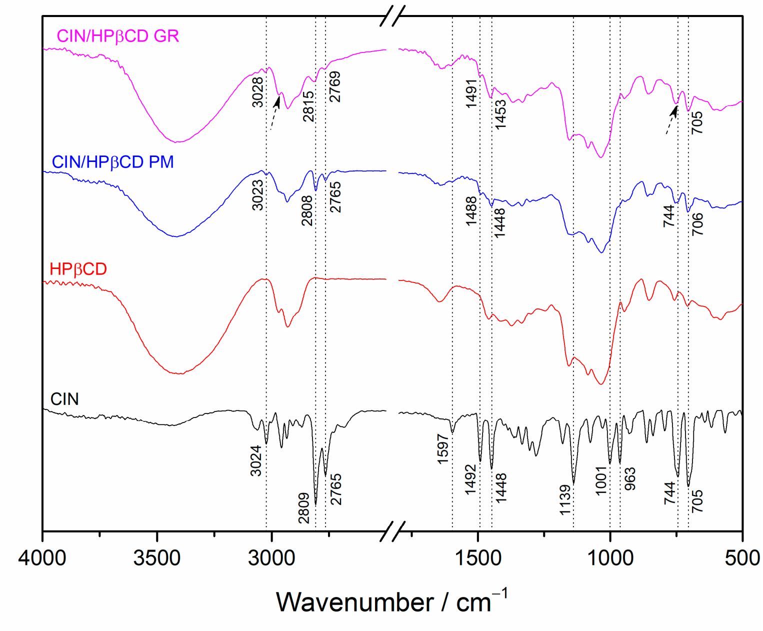

Figure S2. FTIR spectra of pure compounds CIN (–) and HPβCD (–), their physical mixture (–), and co-ground sample prepared at 30 Hz for 40 min (–).

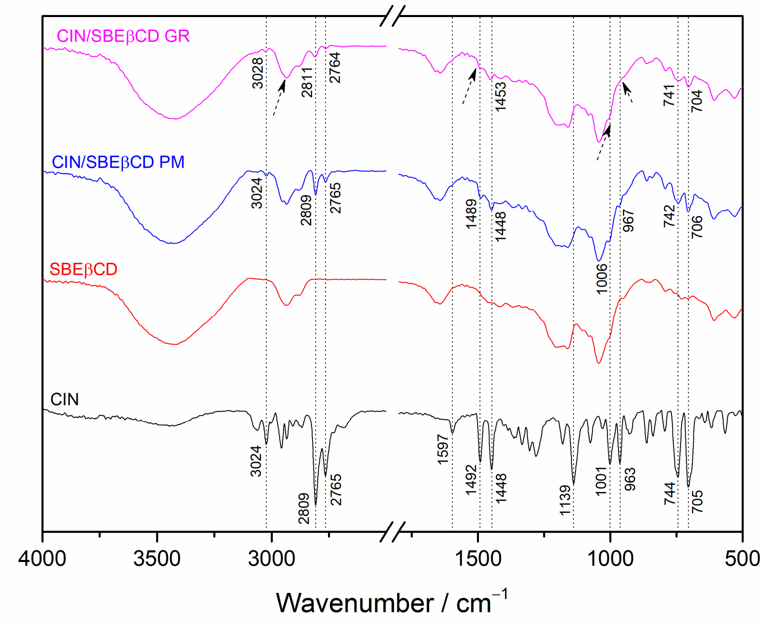

Figure S3. FTIR spectra of pure compounds CIN (–) and SBEβCD (–), their physical mixture (–), and co-ground sample prepared at 30 Hz for 40 min (–).

2.3. In vitro dissolution assessment

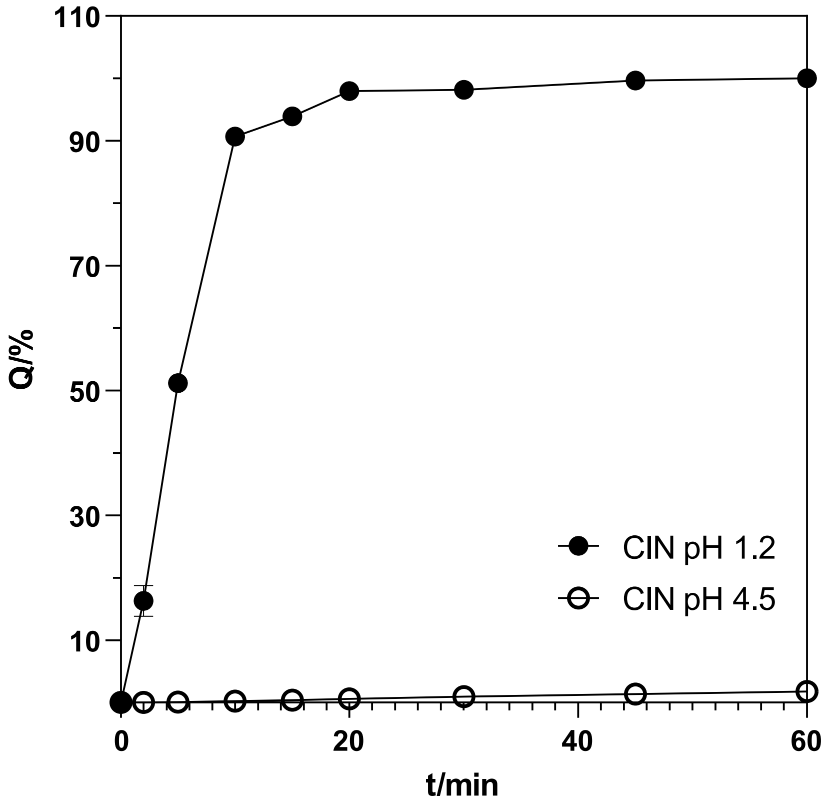

Figure S4. *In vitro* dissolution profiles of CIN in hydrochloric acid medium, pH 1.2, and phosphate buffer solution, pH 4.5, at 37 °C.

3. Effect of β-cyclodextrins on CIN chemical stability

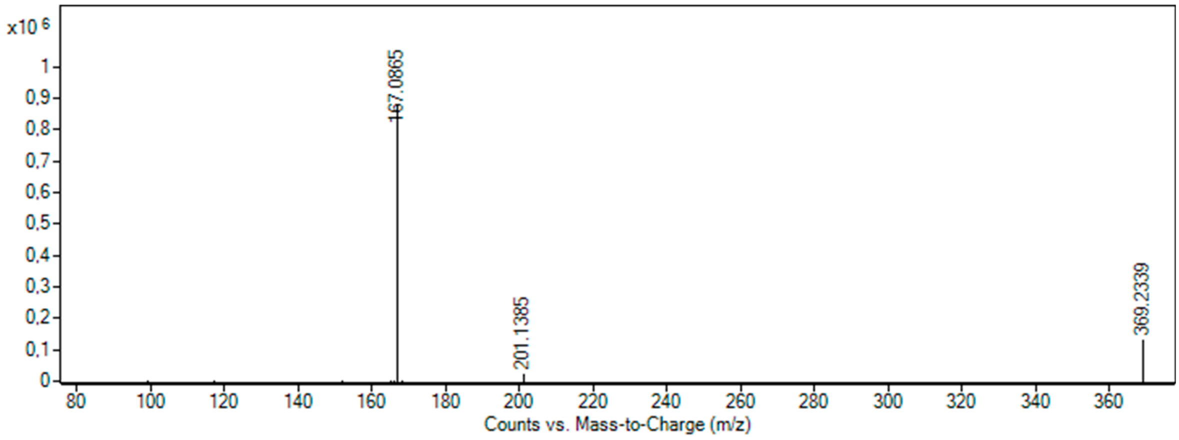

Figure S5. MS/MS spectrum of *m*/*z* 369.2334 (t_R = 7.4 min) at collision potential of 10 V.

Table S7. MS/MS spectra of *m*/*z* 369.2334 (t_R = 7.4 min) at collision potential of 5, 10, 20 and 30 V.

m/*z*	Relative intensity / %				Assignation
	**5 V**	**10 V**	**20 V**	**30 V**	
369.2339	100	14.75	/	/	$[M+H]^+$
201.1385	2.18	2.27	/	/	$[C_{13}H_{17}N_2]^+$
167.0865	56.00	100	100	100	$[C_{13}H_{11}]^+$
152.0618	/	/	/	2.08	$[C_{12}H_8]^+$
117.0699	/	/	/	<1	$[C_9H_9]^+$

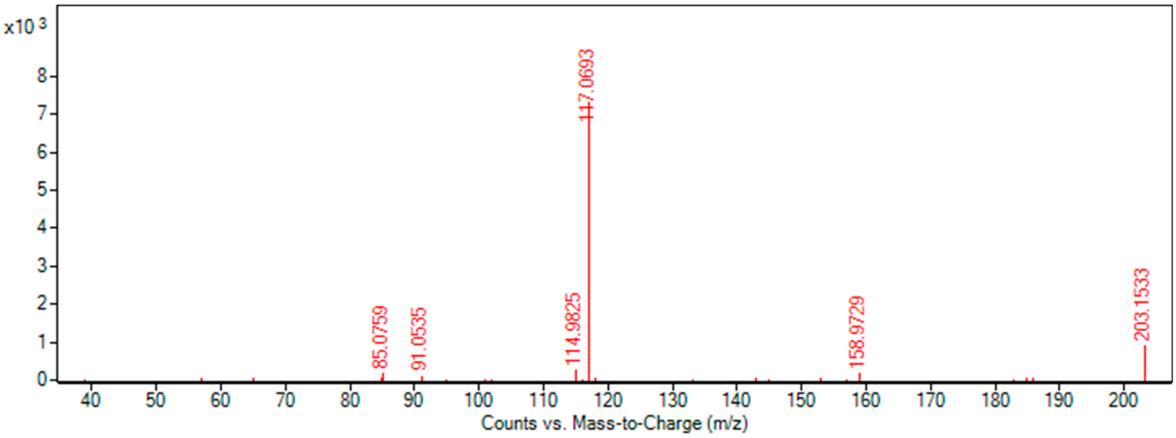

Figure S6. MS/MS spectrum of *m*/*z* 203.1539 (t_R = 7.4 min) at collision potential of 10 V.

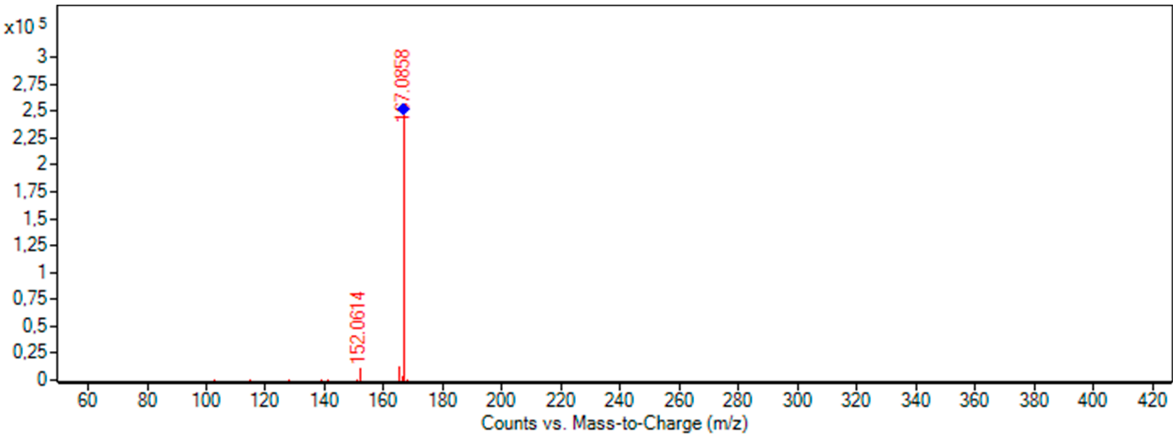

Figure S7. MS/MS spectrum of *m*/*z* 167.0858 (t_R = 7.4 min) at collision potential of 10 V.

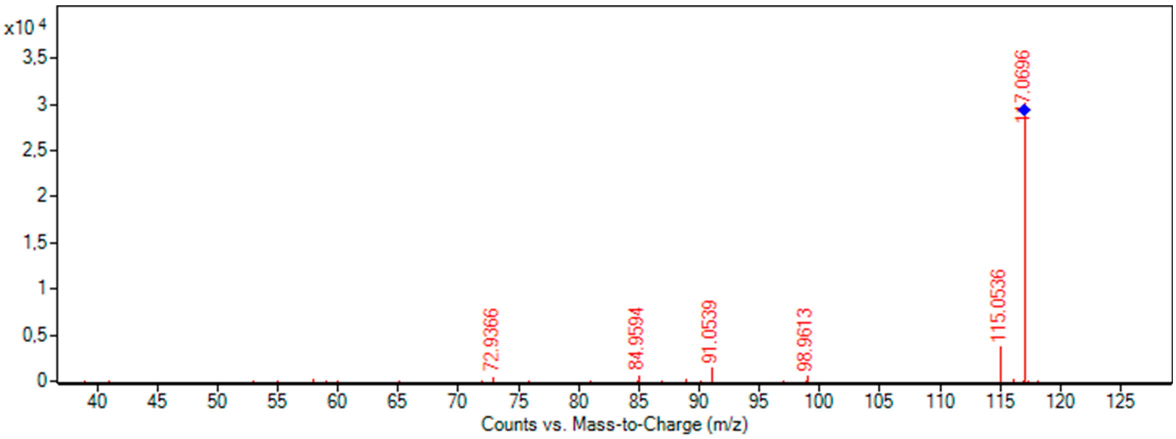

Figure S8. MS/MS spectrum of *m/z* 117.0697 (t_R = 7.4 min) at collision potential of 10 V.

3.1. Hydrolytic acidic degradation

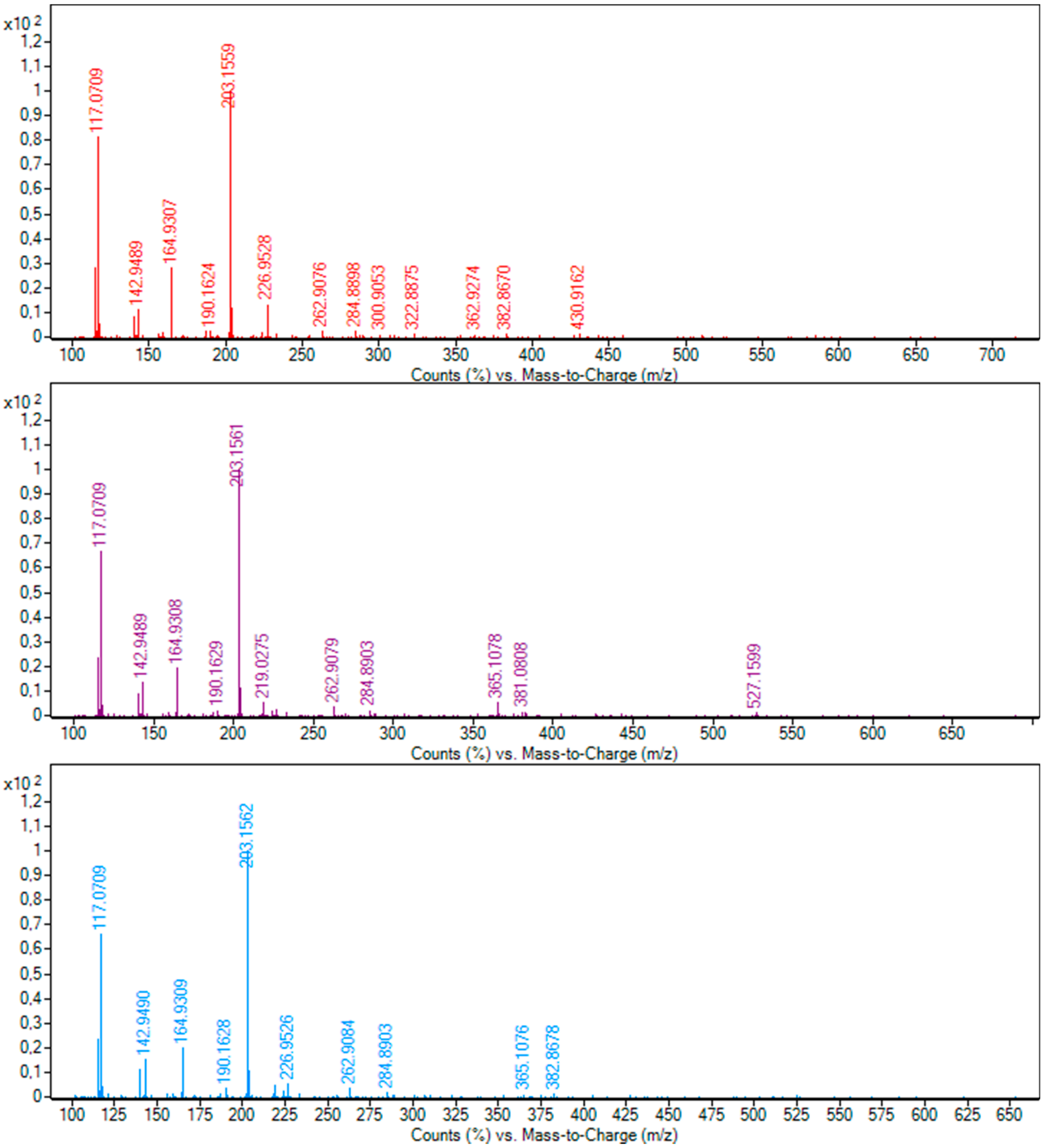

Figure S9. MS spectra at t_R = 0.6 min for the CIN (–), CIN/βCD (–), and CIN/HPβCD (–) samples exposed to acidic forced degradation.

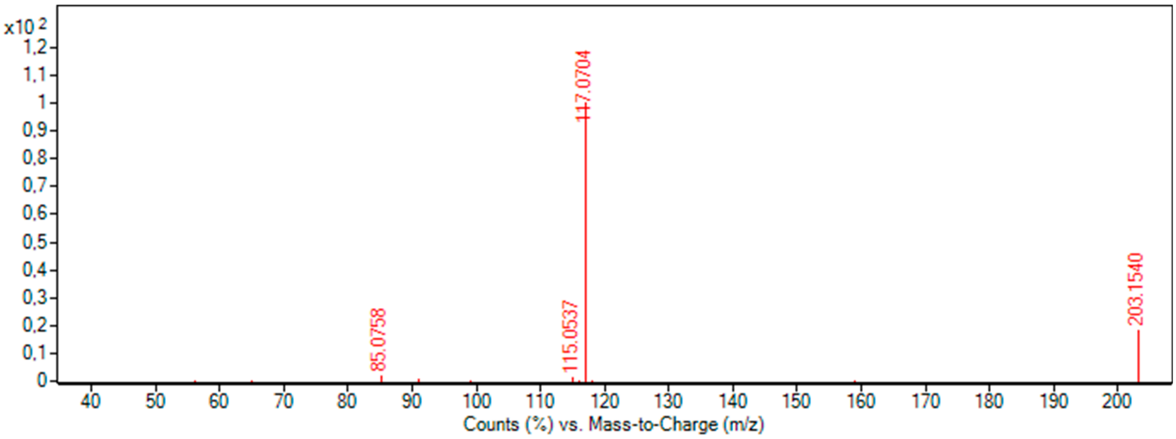

Figure S10. MS/MS spectrum of *m/z* 203.1559 (t_R = 0.6 min) at collision potential of 10 V.

Table S8. MS/MS spectra of *m/z* 203.1559 (t_R = 0.6 min) at collision potential of 5, 10, 20 and 30 V.

m/z	**Relative intensity / %**				Assignation
	**5 V**	**10 V**	**20 V**	**30 V**	
203.1559	100	18.43	/	/	$[M+H]^+$
117.0704	57.79	100	100	100	$[C_9H_9]^+$
91.0542	/	/	1.70	18.42	$[C_7H_7]^+$
85.0758	1.83	1.86	/	/	$[C_4H_9N_2]^+$

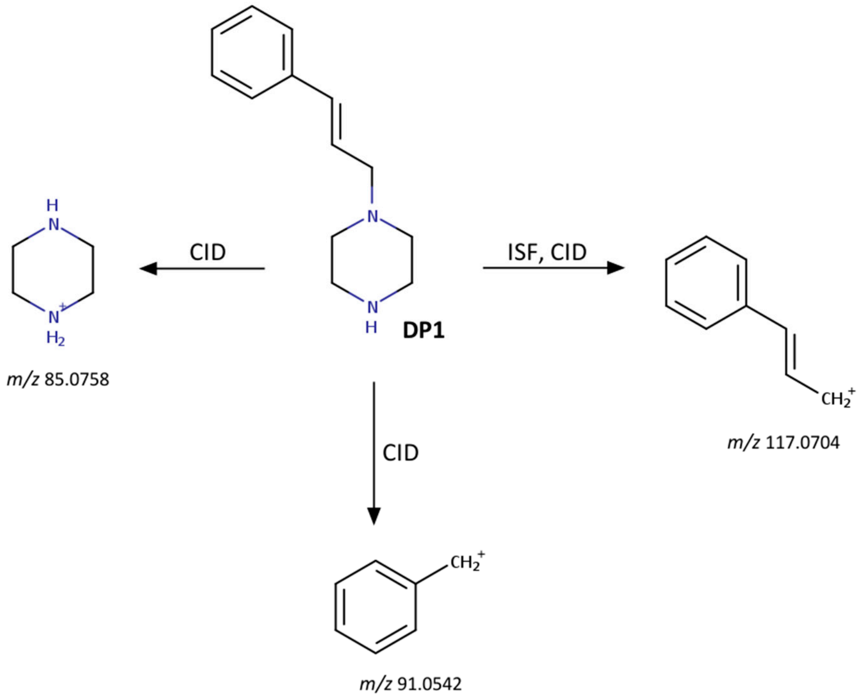

Figure S11. Possible fragmentation pathway of DP1 $[M+H]^+$ ion (*m/z* 203.1559).

3.2. Oxidative degradation

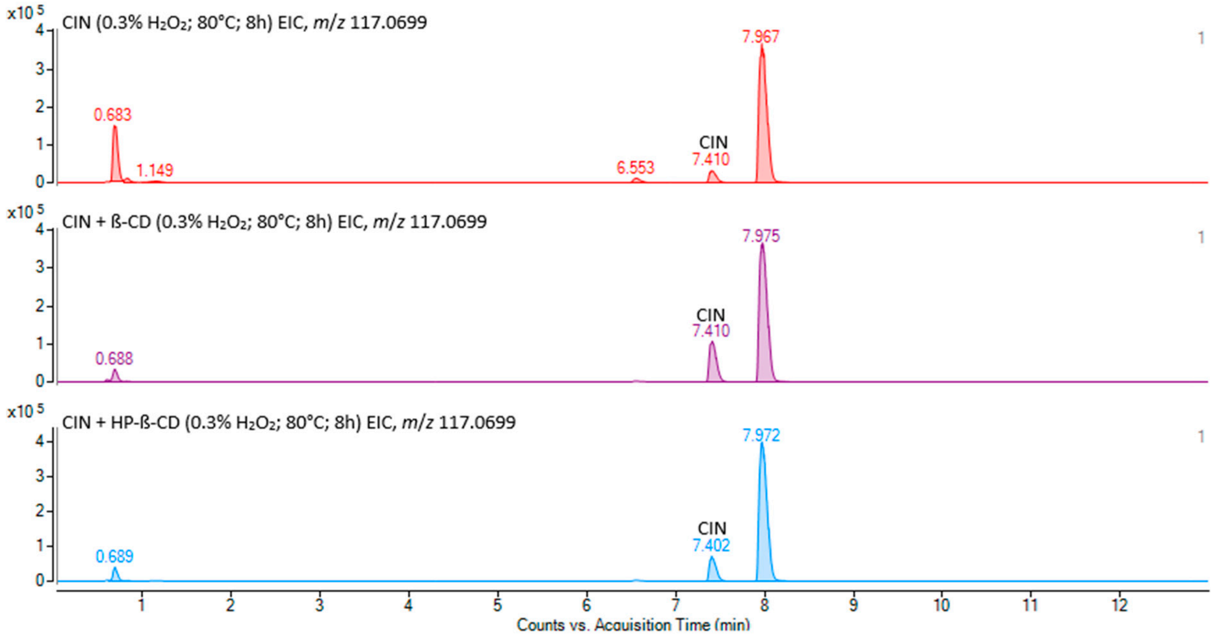

Figure S12. EICs of *m*/*z* 117.0697 marker ion for the CIN (–), CIN/βCD (–), and CIN/HPβCD (–) samples exposed to oxidative forced degradation.

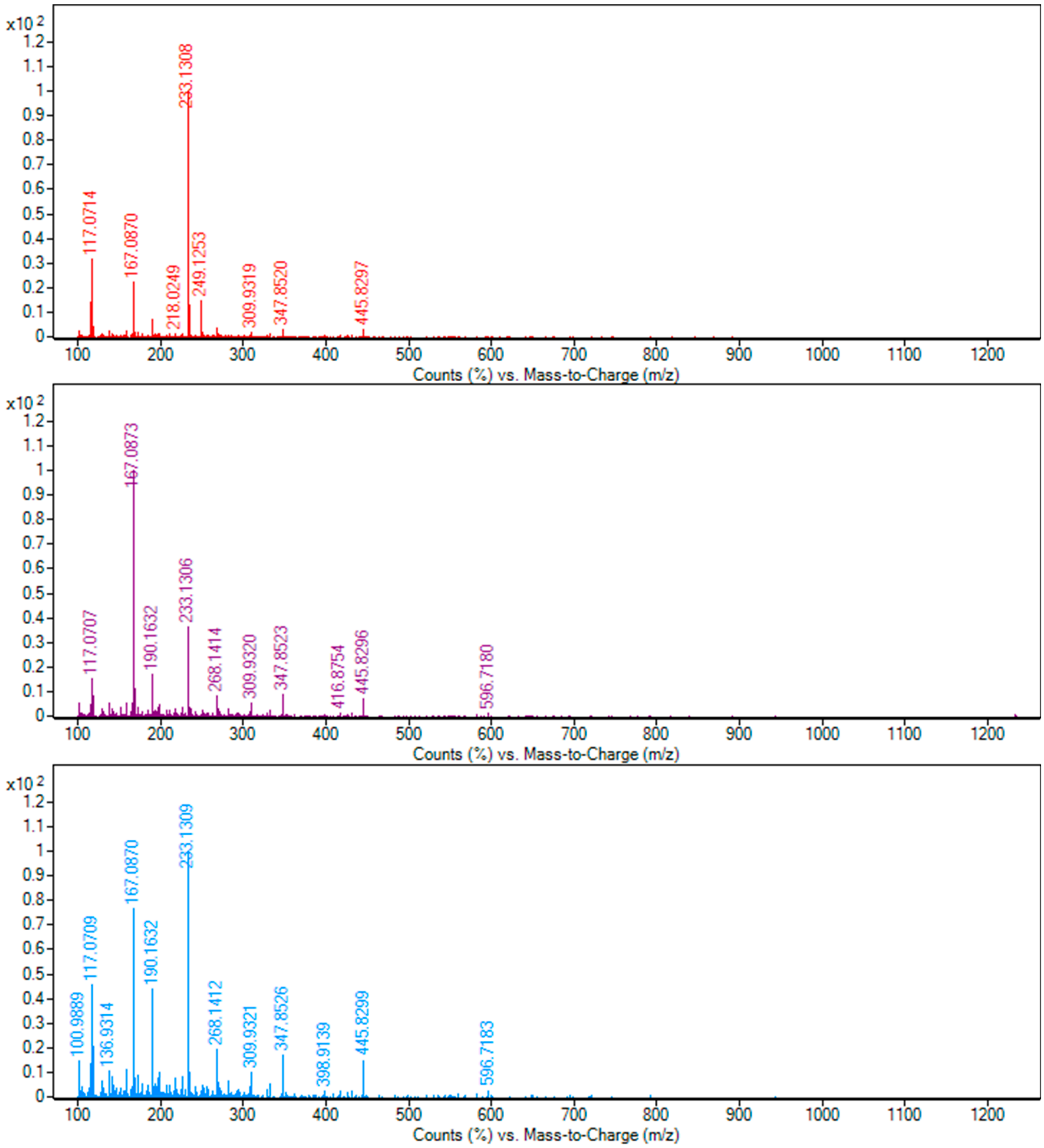

Figure S13. MS spectra at t_R = 0.7 min for the CIN (–), CIN/βCD (–), and CIN/HPβCD (–) samples exposed to oxidative forced degradation.

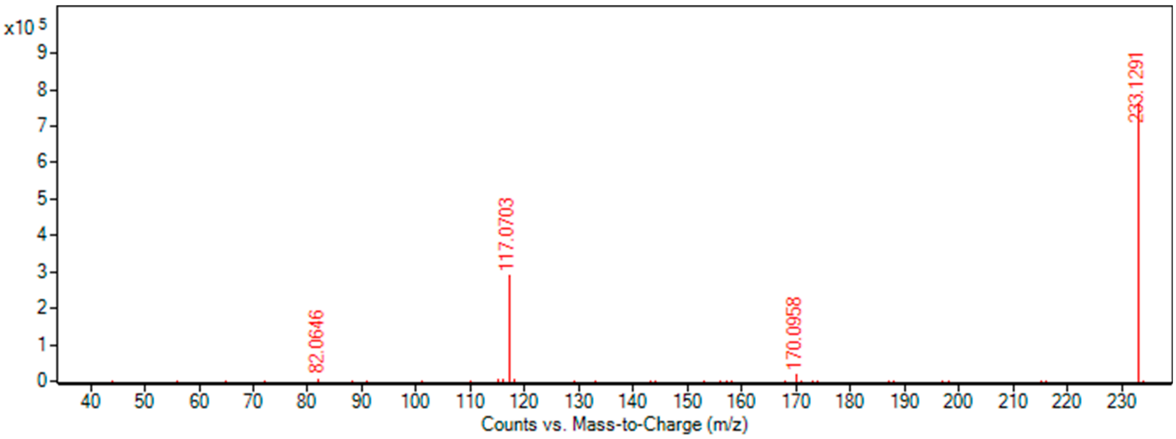

Figure S14. MS/MS spectrum of *m*/*z* 233.1308 (t_R = 0.7 min) at collision potential of 10 V.

Table S9. MS/MS spectra of *m*/*z* 233.1308 (t_R = 0.7 min) at collision potential of 5, 10, 20 and 30 V.

m/*z*	Relative intensity / %				Assignation
	**5 V**	**10 V**	**20 V**	**30 V**	
233.1308	100	100	11.32	/	$[M+H]^+$
117.0703	8.34	38.25	100	100	$[C_9H_9]^+$
91.0544	/	/	1.11	4.55	$[C_7H_7]^+$
82.0646	/	1.04	2.70	3.17	$[C_4H_6N_2]^{•+}$

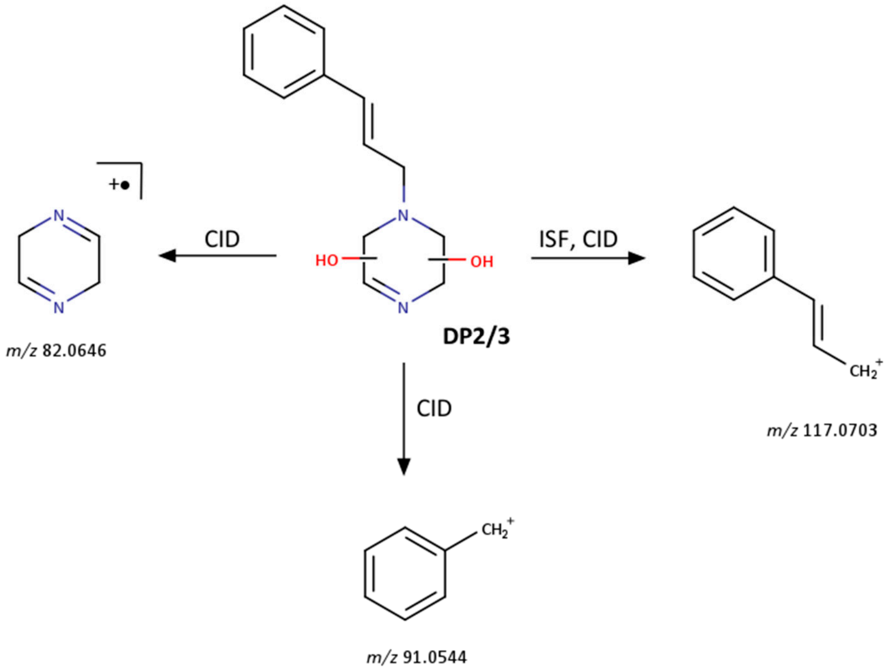

Figure S15. Possible fragmentation pathway of DP2 (and DP3) $[M+H]^+$ ion (*m*/*z* 233.1308).

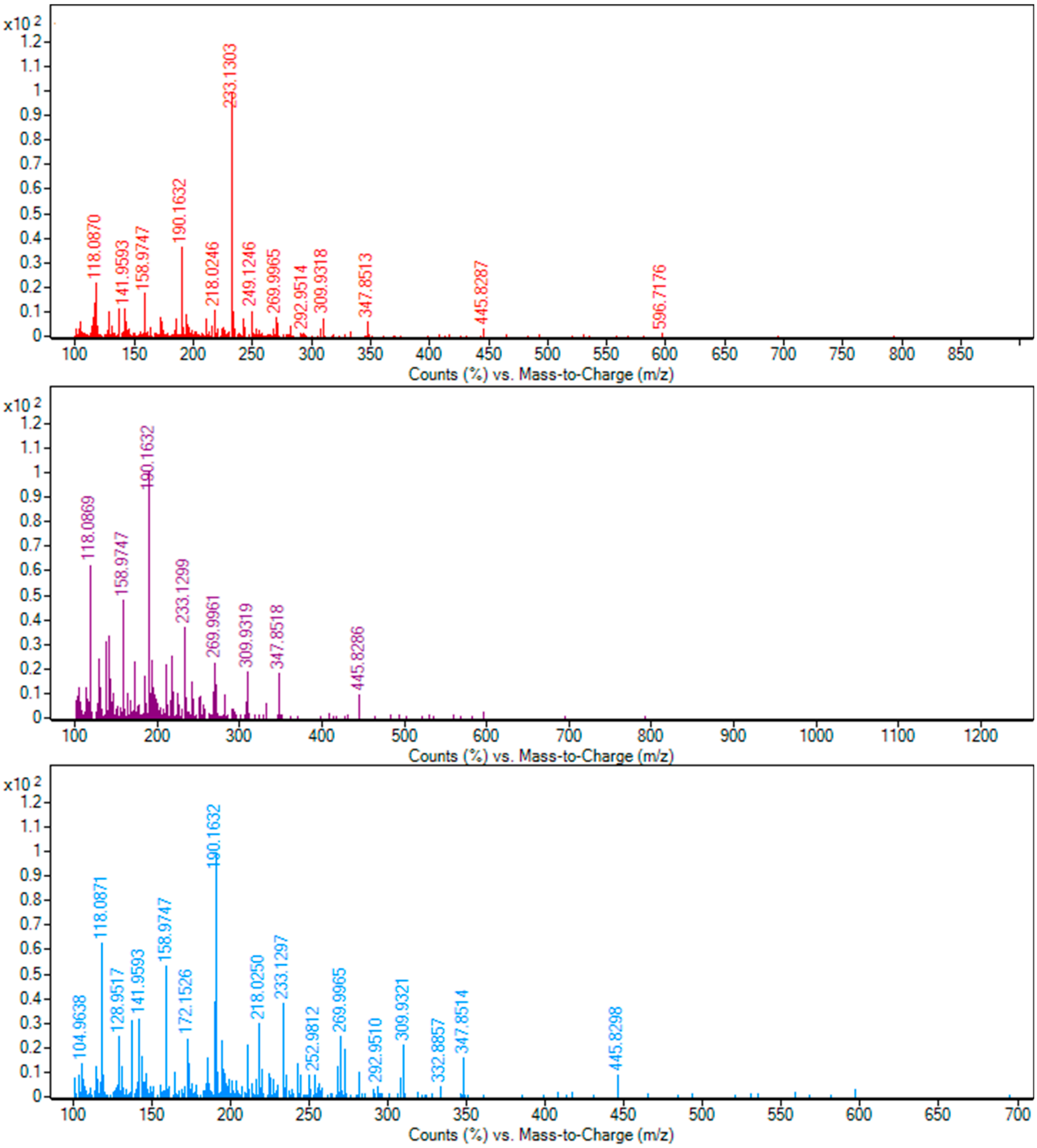

Figure S16. MS spectra at t_R = 0.8 min for the CIN (–), CIN/βCD (–), and CIN/HPβCD (–) samples exposed to oxidative forced degradation.

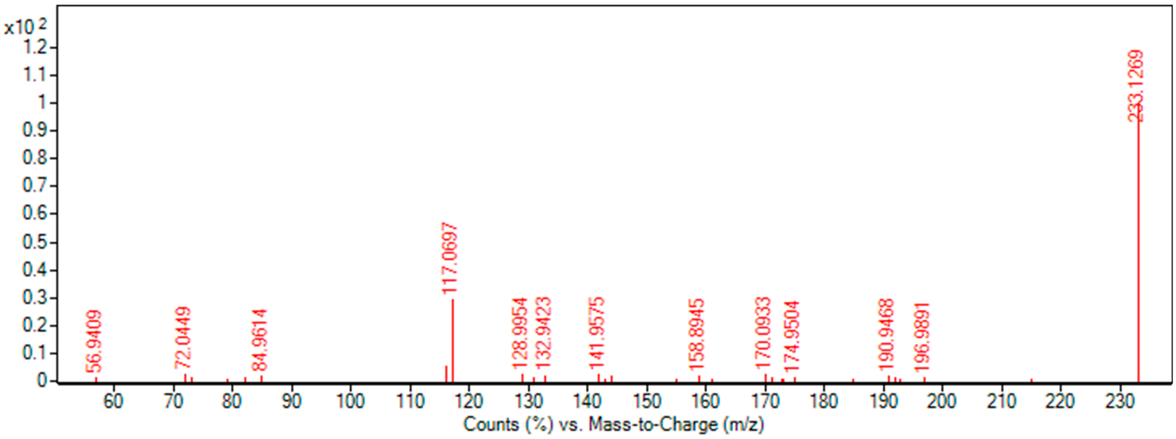

Figure S17. MS/MS spectrum of *m*/*z* 233.1303 (t_R = 0.8 min) at collision potential of 10 V.

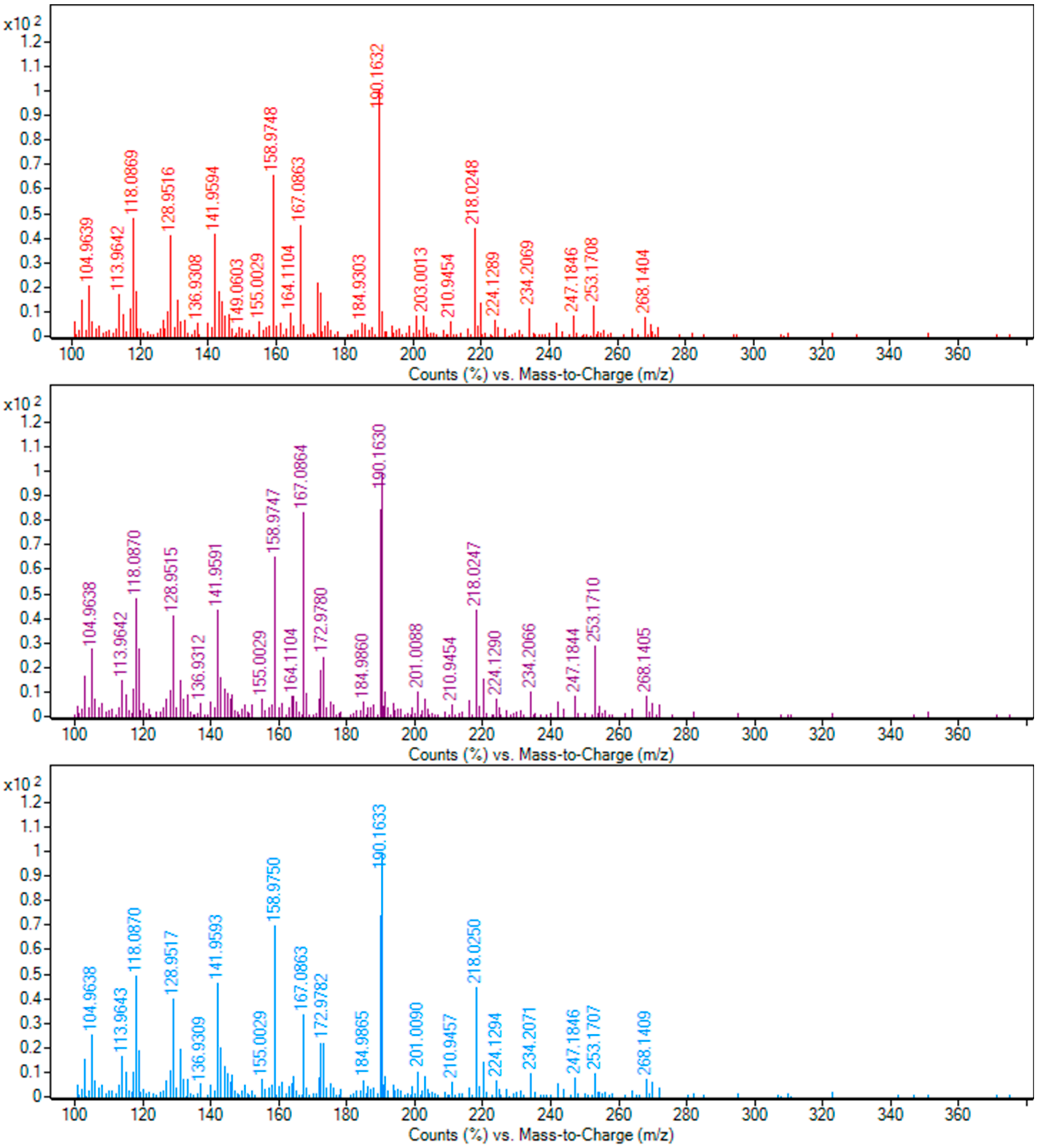

Figure S18. MS spectra at t_R = 4.7 min for the CIN (–), CIN/βCD (–), and CIN/HPβCD (–) samples exposed to oxidative forced degradation.

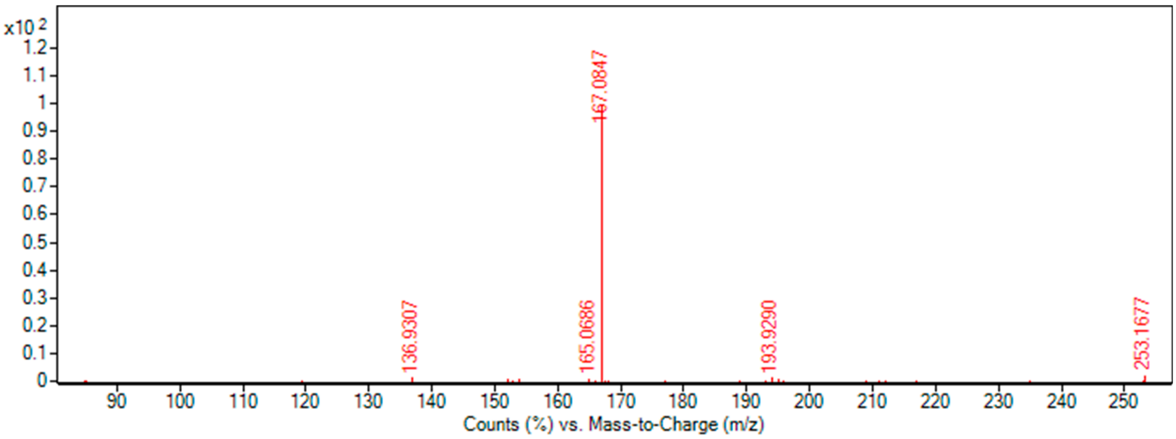

Figure S19. MS/MS spectrum of *m*/*z* 253.1708 (t_R = 4.7 min) at collision potential of 10 V.

Table S10. MS/MS spectra of *m*/*z* 253.1708 (t_R = 4.7 min) at collision potential of 5, 10, 20 and 30 V.

m*/*z	**Relative intensity / %**				Assignment
	**5 V**	**10 V**	**20 V**	**30 V**	
253.1708	35.59	2.27	/	/	$[C_{13}H_{17}N_2]^+$
167.0847	100	100	100	100	$[C_{13}H_{11}]^+$
152.0618	/	/	4.14	26.59	$[C_{12}H_8]^+$

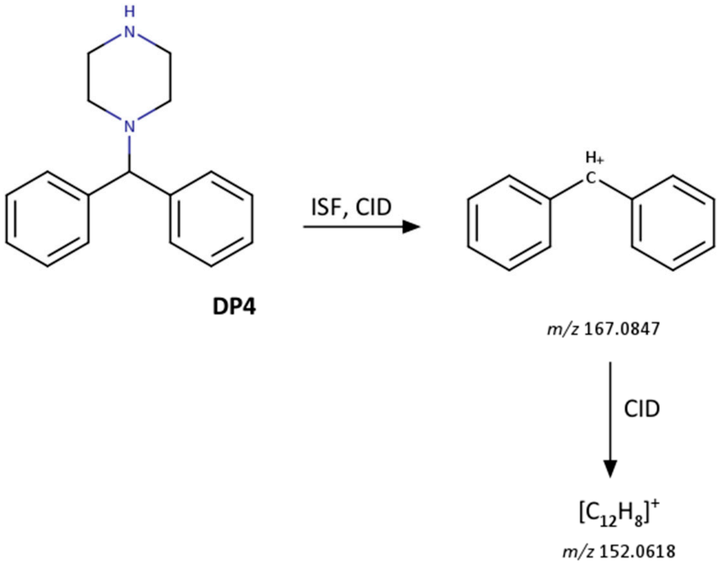

Figure S20. Possible fragmentation pathway of DP4 [M+H]$^+$ ion (*m*/*z* 253.1708).

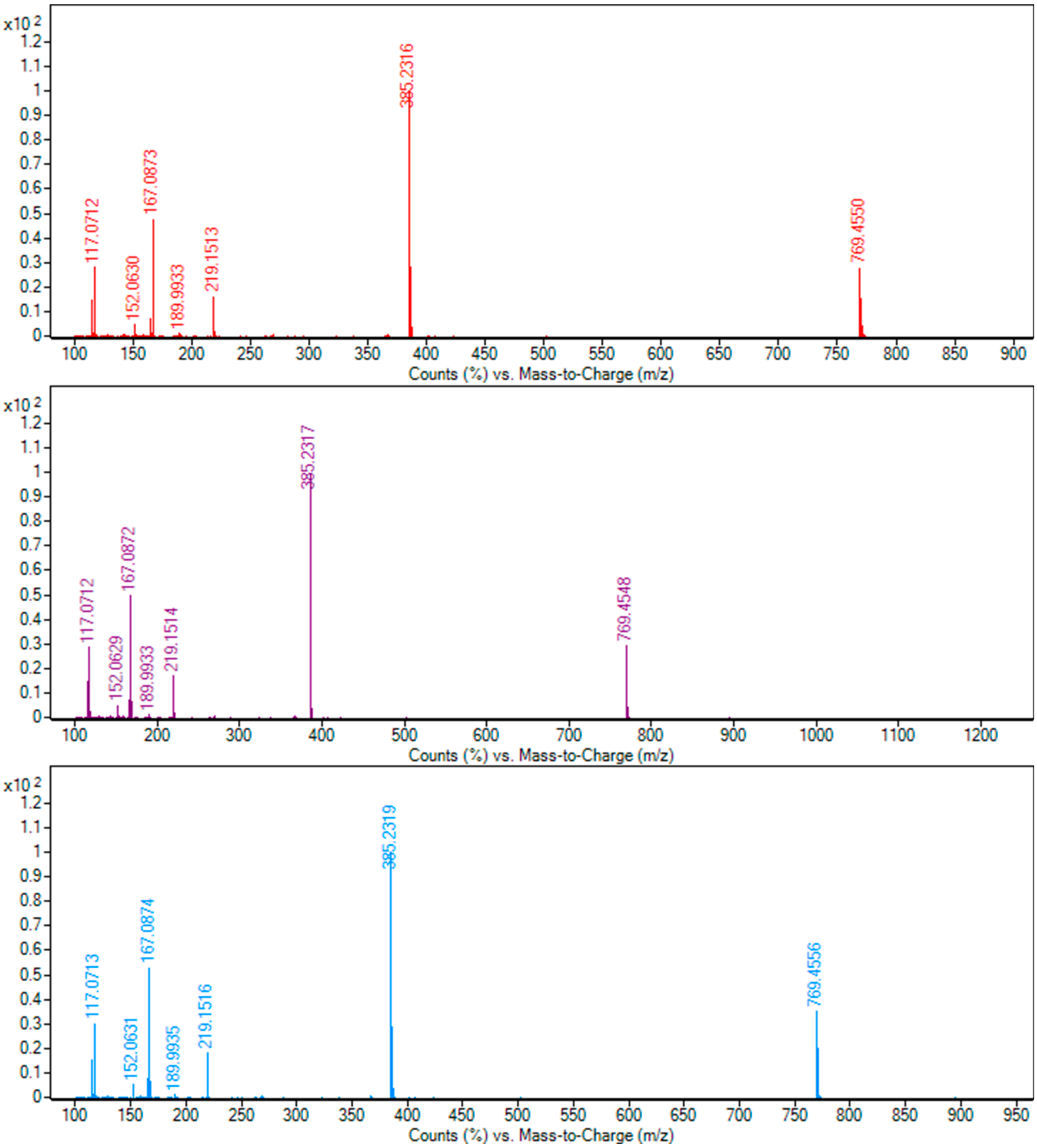

Figure S21. MS spectra at t_R = 8.0 min for the CIN (–), CIN/βCD (–), and CIN/HPβCD (–) samples exposed to oxidative forced degradation.

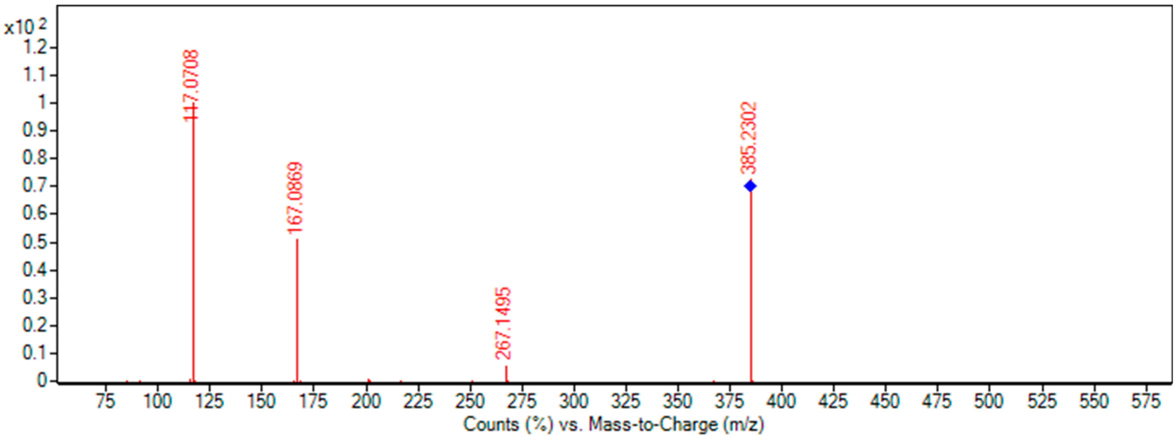

Figure S22. MS/MS spectrum of *m*/*z* 385.2316 (t_R = 8.0 min) at collision potential of 10 V.

Table S11. MS/MS spectra of *m*/*z* 385.2316 (t_R = 8.0 min) at collision potential of 5, 10, 20 and 30 V.

m*/*z	**Relative intensity / %**				Assignment
	**5 V**	**10 V**	**20 V**	**30 V**	
385.2316	100	72.57	/	/	$[M+H]^+$
267.1495	1.42	5.69	/	/	$[C_{17}H_{19}N_2O]^+$
167.0869	10.72	50.91	22.58	18.51	$[C_{13}H_{11}]^+$
117.0708	15.37	100	100	100	$[C_9H_9]^+$
91.0543	/	/	/	1.39	$[C_7H_7]^+$

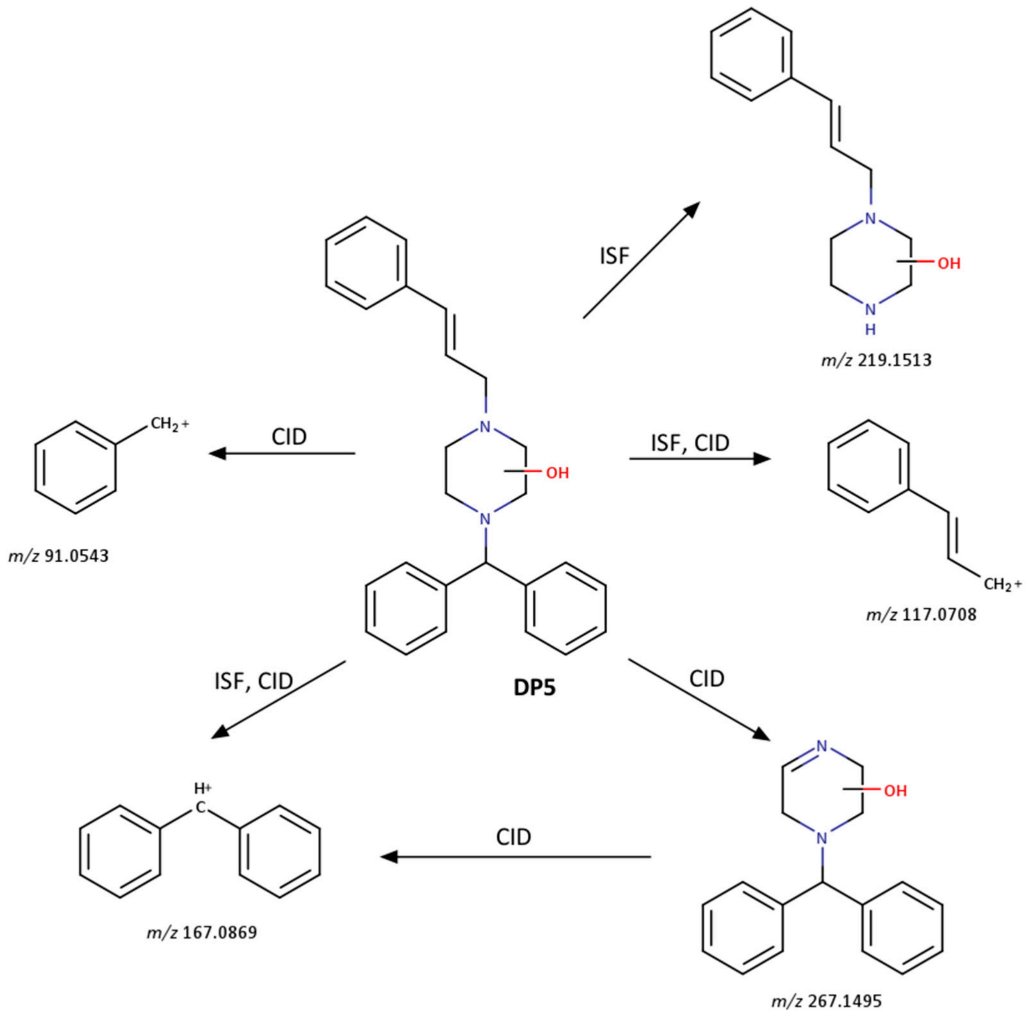

Figure S23. Possible fragmentation pathway of DP5 $[M+H]^+$ ion (*m/z* 385.2316).

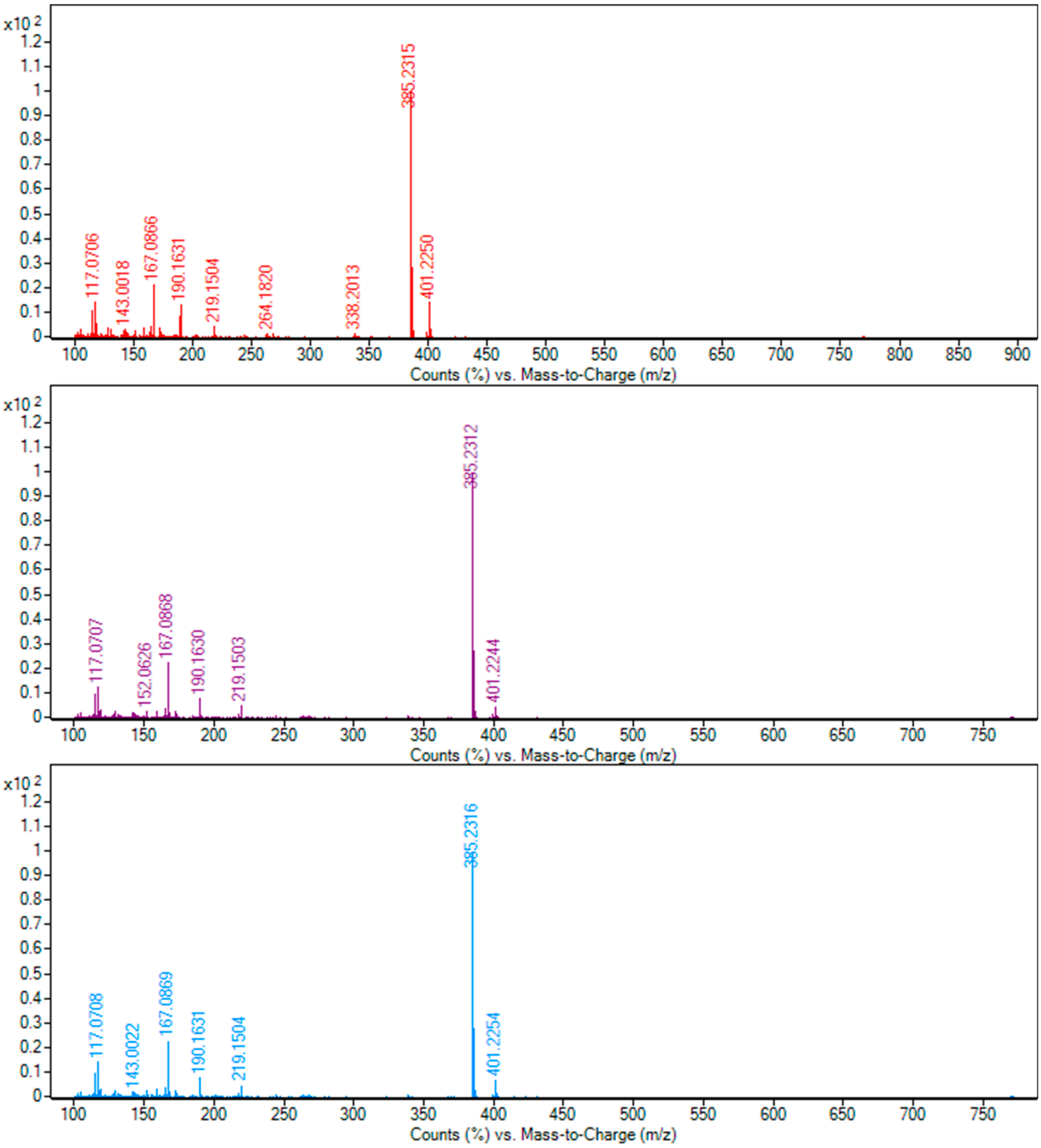

Figure S24. MS spectra at t_R = 8.1 min for the CIN (–), CIN/βCD (–), and CIN/HPβCD (–) samples exposed to oxidative forced degradation.

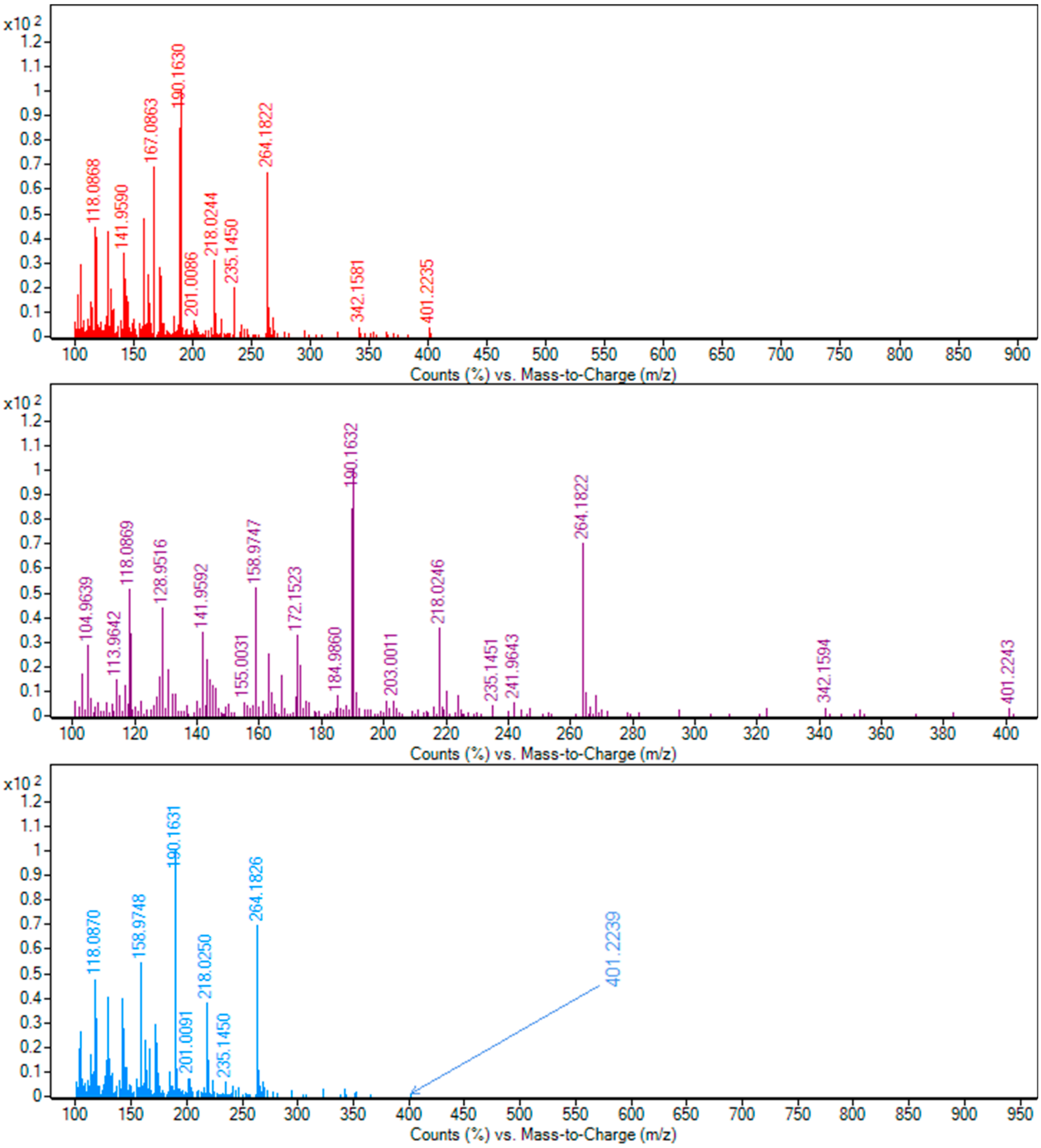

Figure S25. MS spectra at t_R = 6.6 min for the CIN (–), CIN/βCD (–), and CIN/HPβCD (–) samples exposed to oxidative forced degradation; the molecular ion of the DP6 is indicated by an arrow.

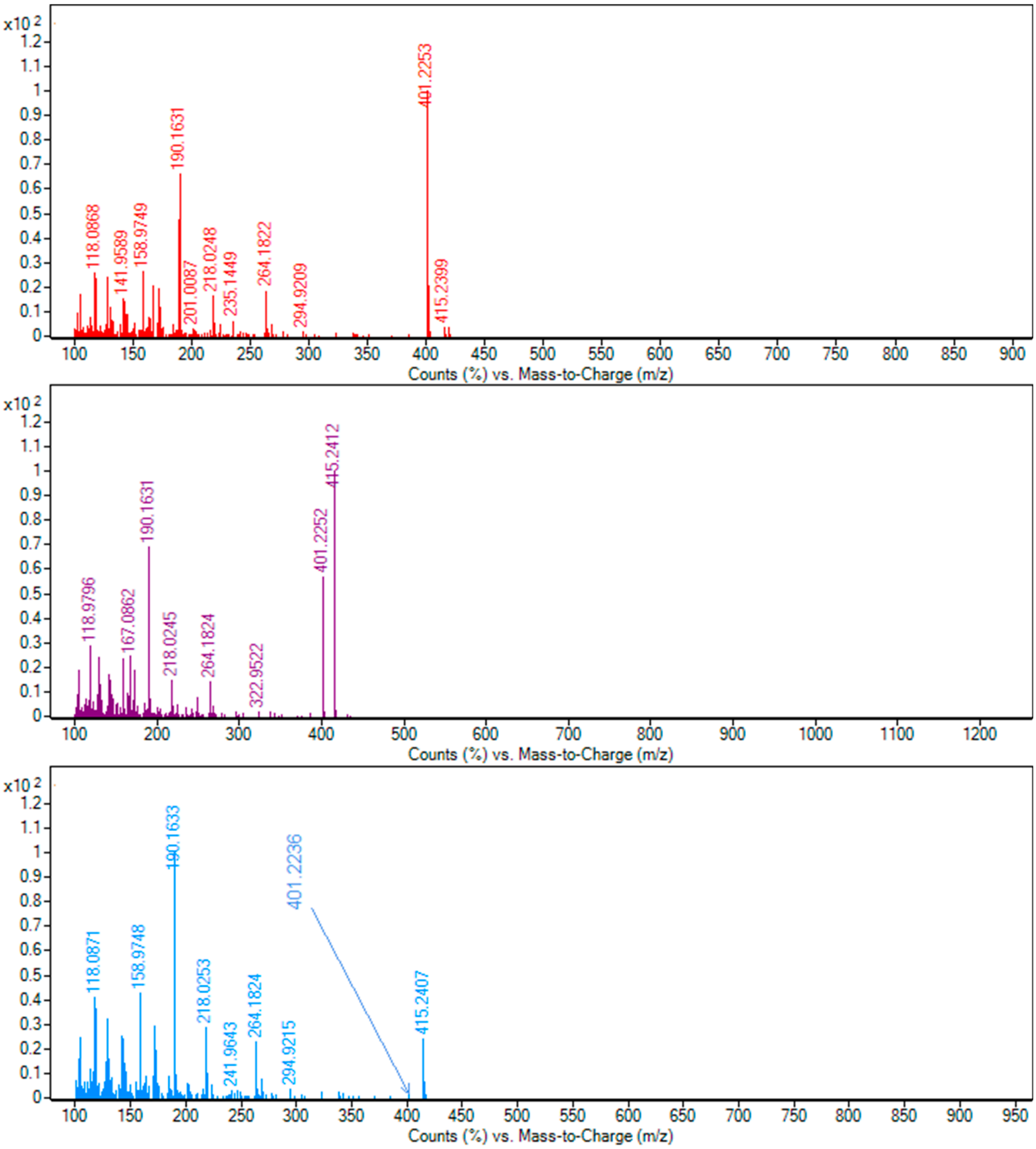

Figure S26. MS spectra at t_R = 7.2 min for the CIN (–), CIN/βCD (–), and CIN/HPβCD (–) samples exposed to oxidative forced degradation; the molecular ion of the DP8 is indicated by an arrow.

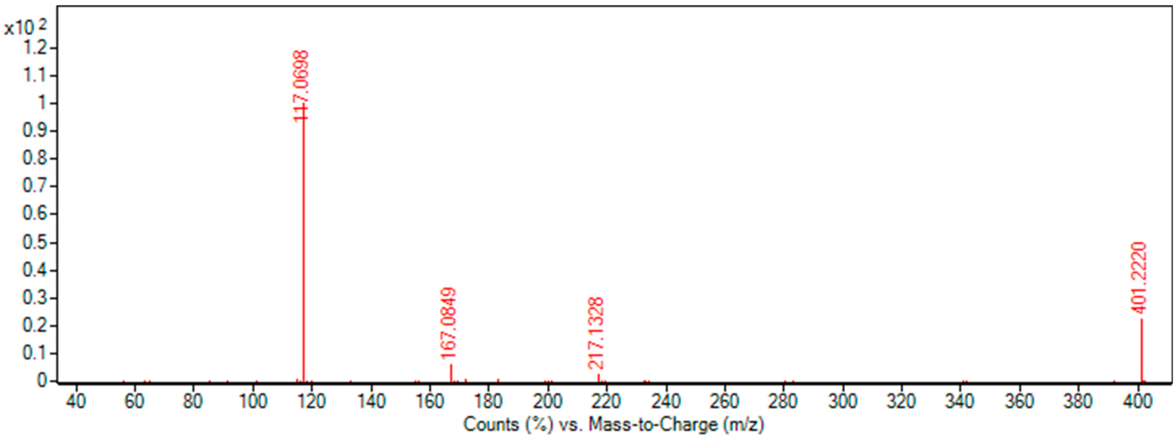

Figure S27. MS/MS spectrum of *m/z* 401.2250 (t_R = 8.1 min) at collision potential of 10 V.

Table S12. MS/MS spectra of *m/z* 401.2250 (t_R = 8.1 min) at collision potential of 5, 10, 20 and 30 V.

m/z	Relative intensity / %				Assignation
	**5 V**	**10 V**	**20 V**	**30 V**	
401.2250	100	22.11	/	/	$[M+H]^+$
217.1328	2.47	2.18	/	/	$[C_{13}H_{17}N_2O]^+$
167.0849	2.63	6.02	2.81	2.61	$[C_{13}H_{11}]^+$
117.0698	36.66	100	100	100	$[C_9H_9]^+$
91.0541	/	/	/	1.74	$[C_7H_7]^+$

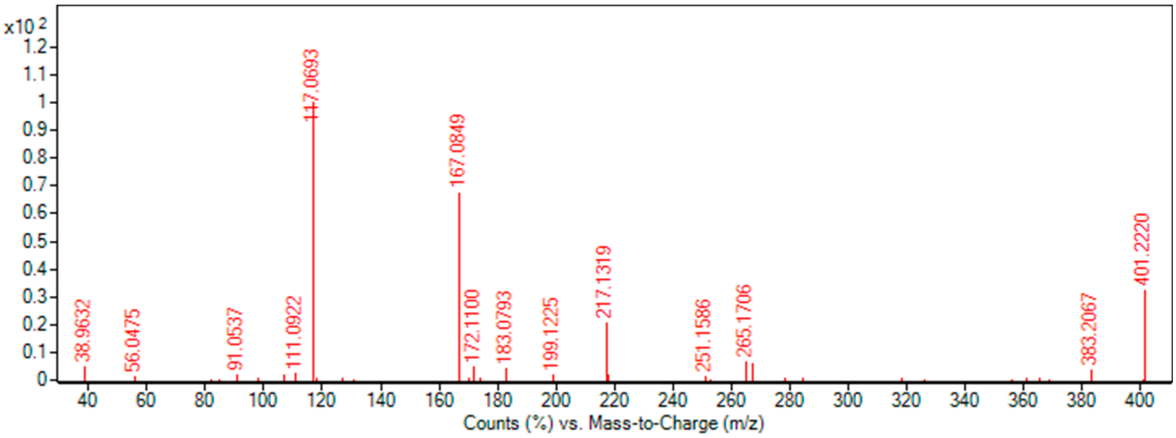

Figure S28. MS/MS spectrum of *m/z* 401.2235 (t_R = 6.6 min) at collision potential of 10 V.

Table S13. MS/MS spectra of *m/z* 401.2235 (t_R = 6.6 min) at collision potential of 5, 10, 20 and 30 V.

m/z	Relative intensity / %				Assignation
	**5 V**	**10 V**	**20 V**	**30 V**	
401.2235	100	32.11	/	/	$[M+H]^+$
217.1319	20.83	20.81	3.78	2.41	$[C_{13}H_{17}N_2O]^+$
167.0849	31.19	67.87	59.84	65.86	$[C_{13}H_{11}]^+$
117.0693	50.21	100	100	100	$[C_9H_9]^+$
91.0537	<1	1.68	/	2.34	$[C_7H_7]^+$

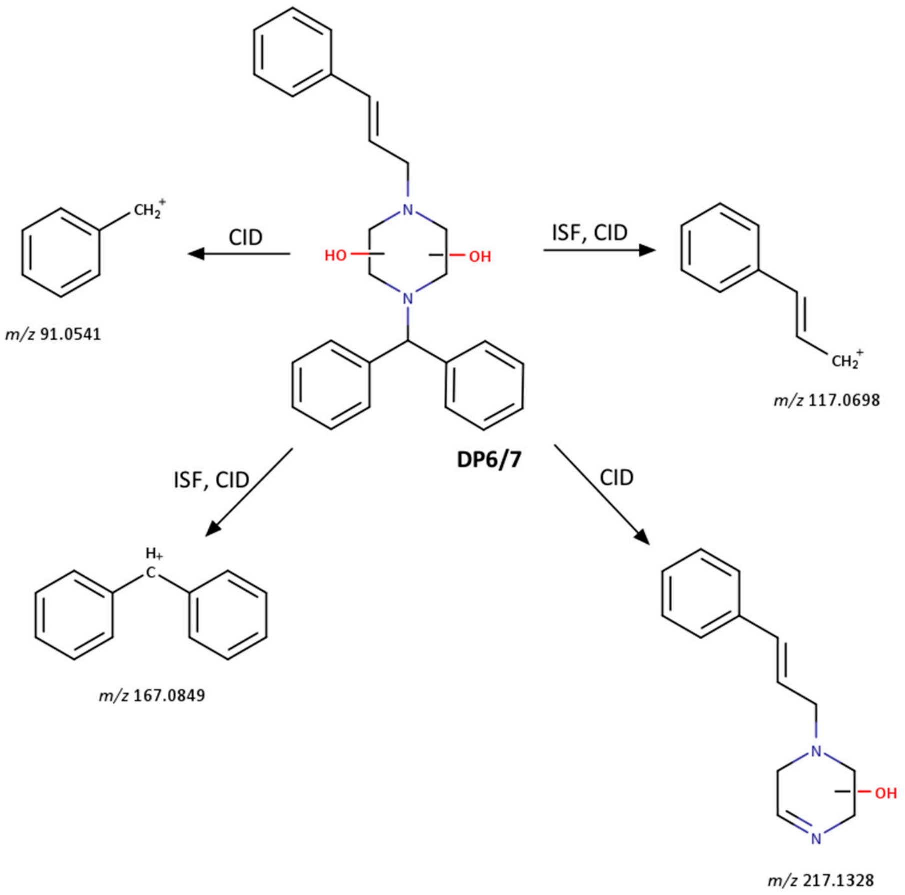

Figure S29. Possible fragmentation pathway of DP6 (and DP7) $[M+H]^+$ ion (*m/z* 401.2250).

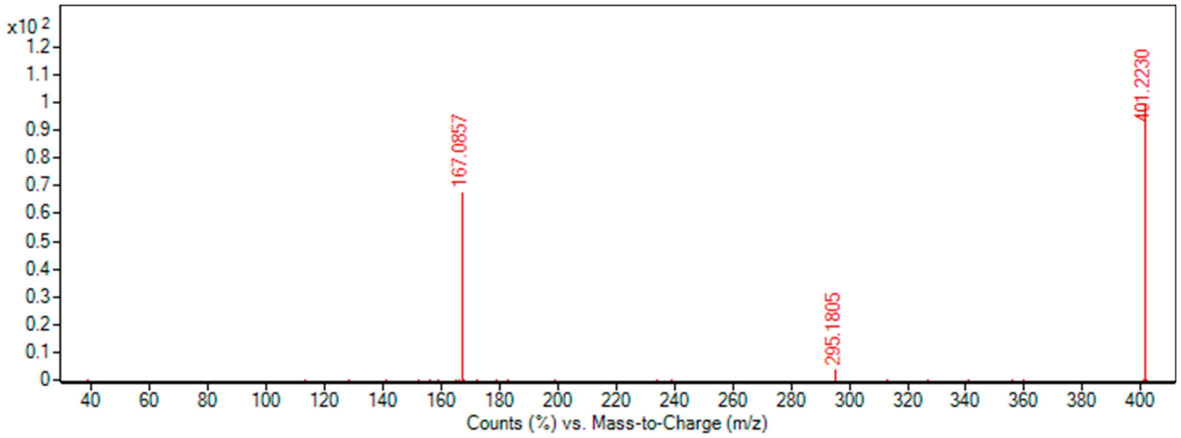

Figure S30. MS/MS spectrum of *m/z* 401.2253 (t_R = 7.2 min) at collision potential of 10 V.

Table S14. MS/MS spectra of *m/z* 401.2253 (t_R = 7.2 min) at collision potential of 5, 10, 20 and 30 V.

m/z	Relative intensity / %				Assignation
	5 V	10 V	20 V	30 V	
401.2253	100	100	1.44	/	$[M+H]^+$
295.1805	/	3.53	1.49	/	$[C_{19}H_{23}N_2O]^+$
167.0857	9.23	67.8	100	100	$[C_{13}H_{11}]^+$
152.0612	/	/	/	2.13	$[C_{12}H_8]^+$

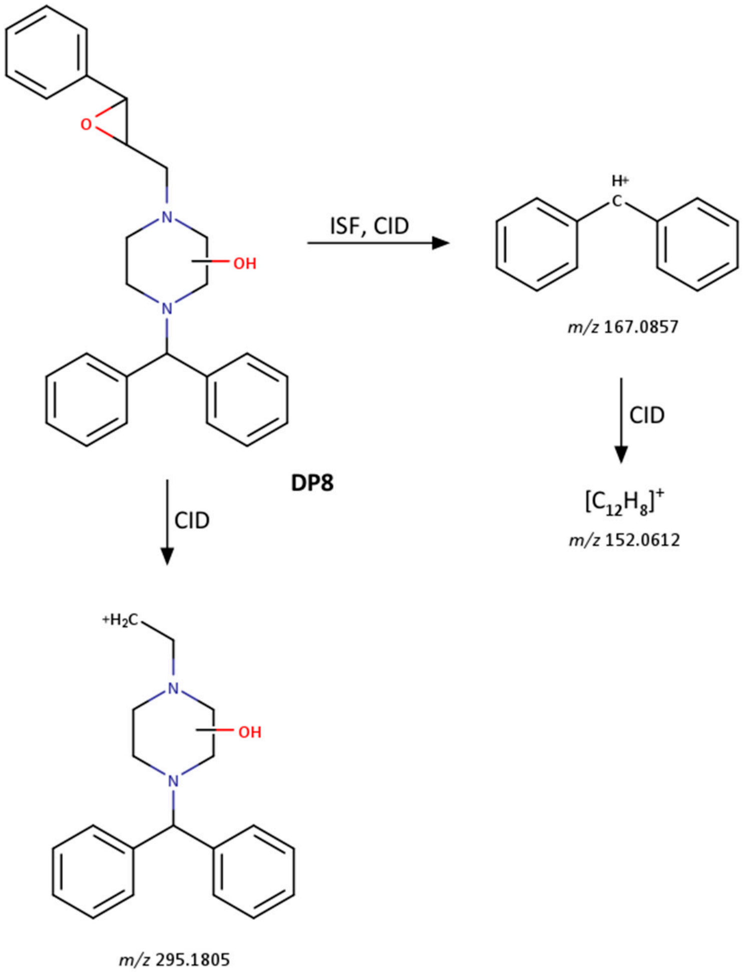

Figure S31. Possible fragmentation pathway of DP8 [M+H]$^{+}$ ion (*m/z* 401.2253).

pharmaceutics

MDPI

4. *In silico* toxicological profiling of CIN degradation products

Table S15. Proposed structures and toxicity data for CIN degradation products.

	Structure	SMILES strings	Toxicity assessment			
			Oral rat LD_{50} (mg/kg)	BCF	DevTox	Mutagenicity
CIN		C(\C=C\C1=CC=CC=C1)N1CCN(CC1)C(C1=CC=CC=C1)C1=CC=CC=C1	433.47	277.22	0.71	0.40
DP1		C(\C=C\C1=CC=CC=C1)N1CCNCC1	329.11	12.10	0.31	0.35
DP2/DP3		OC1CN=CC(O)N1C\C=C\C1=CC=CC=C1	1465.26	N/A	0.65	0.57
		OC1CN(C\C=C\C2=CC=CC=C2)C(O)C=N1	1216.43	N/A	0.59	0.57
		OC1CN=C(O)CN1C\C=C\C1=CC=CC=C1	984.17	6.27	0.59	0.45
		OC1N=CCN(C\C=C\C2=CC=CC=C2)C1O	1706.30	N/A	0.61	0.58
		OC1CN(C\C=C\C2=CC=CC=C2)CC(O)=N1	985.82	5.37	0.62	0.46
		OC1N(C\C=C\C2=CC=CC=C2)CCN=C1O	1051.00	4.76	0.61	0.45
		OC1CN=CC[N+]1([O-])C\C=C\C1=CC=CC=C1	N/A	N/A	0.63	N/A
		OC1C[N+]([O-])(C\C=C\C2=CC=CC=C2)CC=N1	N/A	N/A	0.65	N/A
		OC1=NCC[N+]([O-])(C\C=C\C2=CC=CC=C2)C1	N/A	N/A	0.65	N/A
		OC1C=NCC[N+]1([O-])C\C=C\C1=CC=CC=C1	N/A	N/A	0.63	N/A

DP4	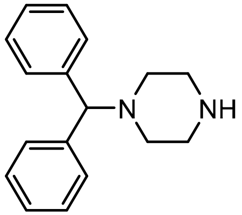	C1CN(CCN1)C(C1=CC=CC=C1)C1=CC=CC=C1	347.30	118.73	0.35	0.46
DP5	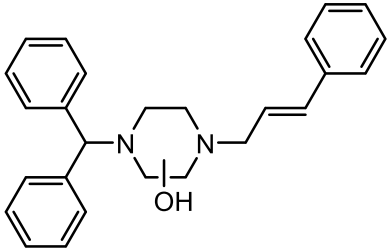	OC1CN(CCN1C\C=C\C1=CC=CC=C1)C(C1=CC=CC=C1)C1=CC=CC=C1	737.20	103.36	0.90	0.53
		OC1CN(C\C=C\C2=CC=CC=C2)CCN1C(C1=CC=CC=C1)C1=CC=CC=C1	717.28	84.45	0.87	0.56
		[O-][N+]1(CCN(C\C=C\C2=CC=CC=C2)CC1)C(C1=CC=CC=C1)C1=CC=CC=C1	372.08	N/A	0.46	N/A
		[O-][N+]1(C\C=C\C2=CC=CC=C2)CCN(CC1)C(C1=CC=CC=C1)C1=CC=CC=C1	387.12	N/A	0.67	N/A
DP6/DP7	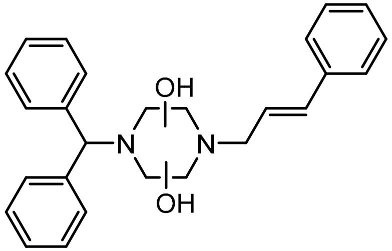	OC1CN(CC(O)N1C\C=C\C1=CC=CC=C1)C(C1=CC=CC=C1)C1=CC=CC=C1	601.46	58.72	0.85	0.37
		OC1CN(C(C2=CC=CC=C2)C2=CC=CC=C2)C(O)CN1C\C=C\C1=CC=CC=C1	511.29	59.14	0.87	0.53
		OC1CN(CC[N+]1([O-])C\C=C\C1=CC=CC=C1)C(C1=CC=CC=C1)C1=CC=CC=C1	548.40	N/A	0.75	N/A
		OC1C[N+]([O-])(CCN1C\C=C\C1=CC=CC=C1)C(C1=CC=CC=C1)C1=CC=CC=C1	508.68	N/A	0.72	N/A
		OC1C(O)N(CCN1C\C=C\C1=CC=CC=C1)C(C1=CC=CC=C1)C1=CC=CC=C1	513.64	52.87	0.83	0.52
		OC1CN(C\C=C\C2=CC=CC=C2)CC(O)N1C(C1=CC=CC=C1)C1=CC=CC=C1	570.91	55.86	0.81	0.56
		OC1CN(C\C=C\C2=CC=CC=C2)CC[N+]1([O-])C(C1=CC=CC=C1)C1=CC=CC=C1	519.95	N/A	0.69	N/A
		OC1C[N+]([O-])(C\C=C\C2=CC=CC=C2)CCN1C(C1=CC=CC=C1)C1=CC=CC=C1	541.58	N/A	0.79	N/A
DP8	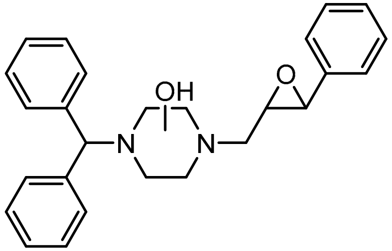	OC1CN(CCN1CC1OC1C1=CC=CC=C1)C(C1=CC=CC=C1)C1=CC=CC=C1	963.29	146.28	0.89	0.16
		OC1CN(CC2OC2C2=CC=CC=C2)CCN1C(C1=CC=CC=C1)C1=CC=CC=C1	869.09	139.18	0.90	0.19
		[O-][N+]1(CCN(CC2OC2C2=CC=CC=C2)CC1)C(C1=CC=CC=C1)C1=CC=CC=C1	377.86	N/A	0.68	N/A
		[O-][N+]1(CC2OC2C2=CC=CC=C2)CCN(CC1)C(C1=CC=CC=C1)C1=CC=CC=C1	385.77	N/A	0.65	N/A
